# Fouling of Reverse Osmosis (RO) and Nanofiltration (NF) Membranes by Low Molecular Weight Organic Compounds (LMWOCs), Part 2: Countermeasures and Applications

**DOI:** 10.3390/membranes15030094

**Published:** 2025-03-17

**Authors:** Yasushi Maeda

**Affiliations:** LG Chem Japan Co., Ltd., Kyobashi Trust Tower 12F, 2-1-3 Kyobashi Chuo-ku, Tokyo 104-0031, Japan; jpmaeda@lgchem.com; Tel.: +81-3-5299-4530

**Keywords:** fouling, organic fouling, internal fouling, flow loss, reverse osmosis, nanofiltration, surfactant, phonolics, plasticizaer, leachables, octanol-water partition coefficient, log P, pore size, SDI, pretreatment, activated carbon, ozonation, AOP, in situ modification, cleaning, CIP, oxidative cleaning, butyl cellosolve, GC-MS, boron, NDMA, urea, IPA, tannic acid, PEG, rejuvenation

## Abstract

Fouling, particularly from organic fouling and biofouling, poses a significant challenge in the RO/NF treatment of marginal waters, especially wastewater. Part 1 of this review detailed LMWOC fouling mechanisms. Part 2 focuses on countermeasures and applications. Effective fouling prevention relies on pretreatment, early detection, cleaning, optimized operation, and in situ membrane modification. Accurate fouling prediction is crucial. Preliminary tests using flat-sheet membranes or small-diameter modules are recommended. Currently, no specific fouling index exists for LMWOC fouling. Hydrophobic membranes, such as polyamide, are proposed as alternatives to the standard silt density index (SDI) filter. Once LMWOC fouling potential is assessed, suitable pretreatment methods can be implemented. These include adsorbents, specialized water filters, oxidative decomposition, and antifoulants. In situations where pretreatment is impractical, alternative strategies like high pH operation might be considered. Membrane cleaning becomes necessary upon fouling; however, standard cleaning often fails to fully restore the original flow. Specialized CIP chemicals, including organic solvent-based and oxidative agents, are required. Conversely, LMWOC fouling typically leads to a stabilized flow rate reduction rather than a continuous decline. Aggressive cleaning may be avoided if the resulting operating pressure increase is acceptable. When a significant flow rate drop occurs and LMWOC fouling is suspected, analysis of the fouled membrane is necessary for identification. Standard FT-IR often fails to detect LMWOCs. Solvent extraction followed by GC-MS is required. Pyrolysis GC-MS, which eliminates the extraction step, shows promise. The review concludes by examining how LMWOCs can be strategically utilized to enhance membrane rejection and restore deteriorated membranes.

## 1. Introduction

The first commercial-scale reverse osmosis (RO) water treatment plant, built in Coalinga, California, in 1965, marked a revolution in water treatment technology [1,2]. This plant’s initial experience with iron hydroxide fouling underscored the critical need for pretreatment and effective operational management. Starting from this early challenge, the subsequent accumulation of operational expertise and continuous research and development focused on mitigating fouling and scaling have been instrumental in the global expansion and refinement of RO technology. Consequently, advancements in effective pretreatment and operation and maintenance (O&M) strategies have been meticulously documented in manuals and publications, providing invaluable resources for RO plant design and operation.

However, the application of RO to treat marginal waters [3,4], such as municipal or industrial wastewater effluents [5], has introduced a new challenge: organic fouling, especially fouling by low molecular weight organic compounds (LMWOCs). The significance of LMWOC fouling is highlighted by the use of nonionic and cationic surfactants as model compounds in evaluating newly commercialized low-fouling membranes [6,7]. This review paper, Part 2, builds upon Part 1, which explains the fouling behavior and mechanisms of LMWOCs [8]. Part 2 summarizes the countermeasures against LMWOC fouling.

Before contextualizing these countermeasures, several manuals and books are reviewed to understand the historical development of fouling prevention strategies. Notably, a 1979 manual detailing measures for preventing fouling and scaling lacked comprehensive strategies for biofouling and omitted information on organic fouling [9]. This lack of information on biofouling likely stemmed from the prevalence of chlorine-tolerant cellulose acetate (CA) membranes at that time. While the description of biofouling has increased significantly in recent manuals [10,11], the section on organic contamination is relatively brief compared to sections dealing with other fouling types, such as particulates, metal oxides, and scaling.

Preventive measures for organic fouling include the following [11]:

- The removal of natural organic matter (NOM), such as humic substances, through coagulation, ultrafiltration (UF), and activated carbon (AC);
- The removal of oils (hydrocarbons or silicone-based) and greases (O&G) using coagulation and AC.

Two additional books elaborate on AC treatment for organic removal [12,13]. However, the specific effectiveness of these treatments in preventing LMWOC fouling remains unclear. Therefore, this review aims to consolidate scattered data and fill the informational gaps present in the existing manuals.

Given that LMWOC fouling is a ubiquitous problem in RO/NF treatment [8,14,15,16,17], careful consideration is crucial during the design and operation of RO/NF plants. Table 1 summarizes corrective and preventive measures specific to LMWOC fouling. While general control strategies, such as pretreatment, detection, cleaning-in-place (CIP), and low-fouling membranes, are similar to those for other fouling types [11,18,19,20], the specific methodologies vary significantly.

This review paper (Part 2) summarizes the reported corrective and preventive measures listed in Table 1. Furthermore, it introduces specific applications, such as rejection enhancement and rejuvenation, that utilize LMWOC foulants.

## 2. Pretreatment

**Figure 1:**
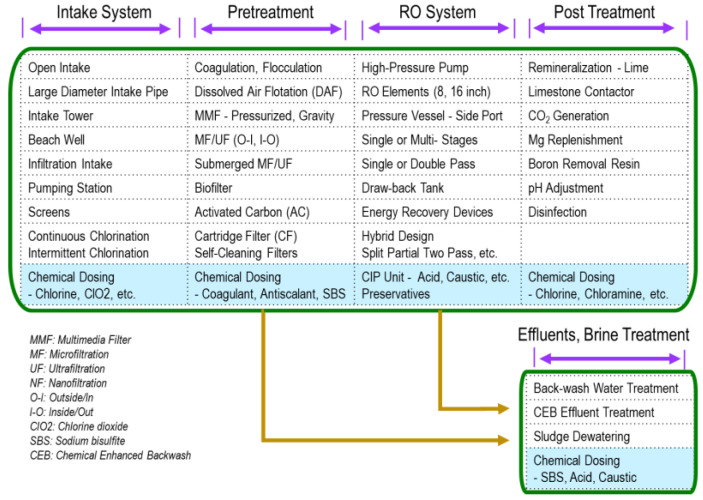
Key unit processes of a seawater desalination RO system and chemical usage. Adapted from Ref. [21].

Pretreatment is one of the essential unit processes in RO/NF plants, as shown in Figure 1 [21]. Potential foulants, such as particulate matter, organics, hardness, etc., can be removed via appropriate pretreatment processes. Chemical addition, including acid or an antiscalant, can prevent inorganic scaling.

However, LMWOCs are more difficult to remove through conventional pretreatment technologies (coagulation/flocculation and media filtration) than high molecular weight organic matters [18]. Fe(III) salt, a common coagulant, is often used to reduce fouling in UF membranes. However, it is not effective in removing LMWOCs [20]. Therefore, to prevent LMWOC fouling, special pretreatment should be considered. The following subsections explain such pretreatment methods.

### 2.1. Adsorption Process

Various methods exist to remove toxic organic compounds from surface water and wastewater. Among those, adsorption is recognized as an effective and low-cost technique for removing organic pollutants and producing high-quality process water [22]. Table 2 shows the technologies used or examined as the adsorption pretreatment.

It has been demonstrated that AC exhibits excellent removal capabilities for most organic pollutants [22]. As mentioned in Part 1, AC has been utilized to investigate the causes of sudden flow rate drops and take corrective actions leveraging its unique characteristics [23,24,25,26]. As a result, it has been proved that the AC process can prevent the flow decline by LMWOCs. AC products come in three forms: granular (GAC), powdered (PAC), and fibrous (FAC). While GAC is commonly used in RO/NF pretreatment, FAC cartridge filters [27] are suitable for bench and pilot studies. The author confirmed that installing an FAC cartridge filter reduced flux decline during full-circulation bench tests. It is also considered effective in preventing LMWOC fouling due to leachables from plant construction components, a concern for new plants. However, FAC has a limited capacity, so GAC is preferable for long-term use.

**Table 2:** Adsorption processes to remove LMWOC foulants in RO/NF.

Adsorbents and Process	Comments	Reference
AC	Granular activated carbon (GAC)	[23,24,25,26]
	Powdered activated carbon (PAC)	[28,29,30,31]
	Fibrous activated carbon (FAC)	[27,32]
AC Material	Bituminous AC	[33,34,35]
	Peat-based AC	[36]
Others	Polyamide (PA)	[37]
	Synthetic adsorbents	[26]
Integrated Process	Coagulation + multi-media filter (MMF) + GAC	[33]
	Coagulation/PAC + MF/UF	[38]
	GAC + UF	[39]
	UF + GAC	[36]
	NF + GAC	[40]
	Membrane bioreactor (MBR) + GAC	[41]
	Advanced oxidation process (AOP) + GAC	[42]

While PAC provides faster adsorption kinetics than GAC, its implementation may be restricted due to handling problems and the difficulty of regeneration [32]. To address handling issues, a hybrid process in which a low-pressure (LP) membrane (MF/UF) is installed to remove spent PAC was disclosed [38]. Shon et al. [28,29,30,31] demonstrated that pretreatment involving flocculation (with FeCl_3_) followed by PAC adsorption significantly improved the prevention of RO/NF membrane normalization flux decline in biologically treated sewage effluent. The flocculant and PAC mainly remove the hydrophobic portion of EfOM and the majority of LMWOCs [29]. A PAC dose of 0.5 g/L was sufficient to remove most 300–5000 Da organics. The effect of the PAC has been confirmed not only in RO/NF but also in the UF process [43]. A significant decrease in the UF membrane fouling was observed by PAC dosing. This could be beneficial for alleviating both UF and RO fouling when an integrated treatment process (PAC–UF–RO) is applied to wastewater with a high organic load.

Regarding the raw materials of AC, it was reported that stiffer and more dense bituminous-based media exhibit better performance than lignite media [34,35]. Bituminous-based AC is less susceptible to attrition and provides better TOC reduction. In the column test using secondary effluent with 18.9 mg/L TOC, the following effluent TOC data were obtained: 4.1 mg/l for the lignite-based AC and 2.1 mg/L for the bituminous-based AC [33]. Peat-based AC also has excellent adsorption capability of surfactants [36].

To improve the performance of AC processes, which suffer from problems like fine particle generation, capacity limitations, and biofouling, various integrated processes have been proposed (Table 2). These integrated processes are implemented depending on the raw water characteristics:

- When raw water has high turbidity or suspended solids, pretreatment with coagulation/filtration or MF/UF is used to prevent AC clogging.
- For feed water with high TOC concentrations, integrated processes—including MBR, NF, and AOP—are utilized to extend AC lifespan by removing organic compounds. For example, alum clarification applied to secondary effluent treatment has been shown to increase TOC removal in solids contact clarifiers to 30–45%, leading to a prolonged carbon life [33].

Several examples illustrate the specific benefits of integrated water treatment processes. For instance, UF demonstrates superior fouling reduction compared to an MF–GAC process [36] owing to its ability to remove colloids and polymeric organic substances. In a refinery wastewater reuse system, Wang et al. [39] highlighted the necessity of placing a GAC tower before the UF unit. This pre-UF GAC step effectively removed LMWOCs that would otherwise foul the RO membrane if UF was used alone, while simultaneously minimizing fine GAC particle penetration and reducing biofouling potential. Furthermore, a pilot-scale MBR–GAC integrated process showed that GAC pretreatment resulted in 80–90% dissolved organic matter (DOM) removal from MBR effluents, stabilizing RO membrane permeability and enhancing permeate quality [41].

An alternative approach using NF instead of MF/UF has been proposed [40]. As described in Part 1, piperazine polyamide (PPA) has a less fouling nature for surfactants, etc., so NF–GAC can be used for the pretreatment of RO. Since NF effectively removes organic matter, only the minimal removal of LMWOCs with GAC is required. The same effect can also be anticipated for AOP-treated water with the added advantage of removing residual oxidants like ozone [42].

AC is widely used for the adsorptive removal of organic substances but has several drawbacks. The regeneration of AC is costly, energy-intensive, and results in a high attrition rate. Additionally, AC tends to adsorb most organic chemicals indiscriminately, making it difficult to selectively recover specific organic chemicals for reuse [44]. To overcome these limitations, alternative adsorption processes using synthetic adsorbents have been proposed. For example, polypropylene (PP) oil adsorbents have been investigated for oil spill cleanup [45]. These PP adsorbents effectively reduced oil concentrations from 4–8 mg/L to below the target level of 1 mg/L within 20 days. Synthetic adsorbents are typically composed of spherical, crosslinked polymer particles with a porous structure [46]. Hydrophobic macroporous styrene–divinylbenzene copolymers are the most commonly used [44]. These synthetic adsorbents can efficiently adsorb and remove bisphenol A, a potent foulant of RO/NF membranes. Hitotsuyanagi et al. [26] conducted RO tests using treated effluent from a machinery factory, comparing GAC and a synthetic adsorbent. Both GAC and the synthetic adsorbent reduced fouling, but no significant difference in their effectiveness was observed. Furthermore, PA, the material constituting the active layer of TFC RO membranes, has also been proposed as an adsorbent. [37].

### 2.2. Specialty Water Filters

Cartridge filters typically need to be replaced every 4 to 8 weeks [47]. Thus, if specialty filters can remove LMWOCs and last for a comparable period, the filters could be applied to RO/NF processes. The application of FAC cartridge filters has already been mentioned. A pilot study evaluating the capability of PP and polyolefin cartridge filters to remove crude oil contaminants from seawater indicated that the short-term performance of an RO element was not significantly affected by the oil-contaminated feedwater [48]. The water analysis results indicated that the filter sets would retain almost all the crude oil. However, the PP-based cartridge filters cannot fully remove LMWOC foulants. Thus, some modifications or other types of materials are necessary.

One such modified filter, MYCELX, was coated with a patented polymeric surfactant technology to enable the removal of oil droplets [49]. MYCELX filters are considered effective in removing hydrocarbons, oil sheen, synthetic oil, and natural oil [50]. The MYCELX filters applied to protect RO membranes from oil fouling reduced the inlet water concentration of 5 ppm of oil and grease (O&G) to less than 0.3 ppm [51].

While not categorized as cartridge filters, MF/UF membranes with layer-by-layer (LbL) formed PEC were reported to be effective at removing organic foulants from a machine shop’s wastewater effluent, preventing the initial flow decline of the TFC PA membrane [52]. To confirm its effectiveness, a cellulose acetate (CA) RO membrane was also tested and compared. Notably, the use of the CA RO membrane as a prefilter did not effectively prevent LMWOC fouling. The PEC MF/UF membranes may be prepared by using anionic-charged membranes as a base membrane, e.g., sulfonated polyether sulfone (PES), hydrolyzed polyacrylonitrile (PAN) [53,54], etc. The PEC layer is formed by simply contacting with polycation solutions.

### 2.3. Oxidation and AOPs

The direct method for removing LMWOCs is oxidative decomposition. UV, chemical, and ozone oxidation have been widely used in water treatment. AOPs have been attracting particular attention as a method for the oxidative decomposition of refractory organic matter. AOPs are a set of chemical treatment procedures, e.g., UV/hydrogen peroxide (H_2_O_2_), UV/Cl_2_, ozone (O_3_)/UV, O_3_/H_2_O_2_, etc., that can remove organic materials by oxidation reactions with a hydroxyl radical (OH) [55]. The concept has been extended to the oxidative processes with sulfate radicals (SO_4_^−^) [56]. Table 3 shows the oxidation treatment technologies considered pretreatment for organic removal. Various AOPs have been proposed and applied to wastewater treatment; ozone-based systems are predominantly considered for the pretreatment of RO/NF.

Chlorine and potassium permanganate (KMnO_4_) have been used to oxidize inorganics such as Fe(II), Mn(II), and As(III) to prevent metal oxide fouling or improve rejection. While chlorine is common, its use for removing organics in RO/NF pretreatment is limited. Sugita et al. [57] investigated oxidation using chlorine to reduce the biofouling and organic fouling of RO in the MBR/RO process. They reported that fouling could be reduced by adding 10 mg/L sodium hypochlorite and setting the residence time at 30 min. Furthermore, adding an acid shock (once a day for 1 h, pH 3) could suppress the pure water permeability coefficient decrease to −2.5%/month. Three-dimensional excitation-emission matrix (EEM) fluorescence spectra analysis revealed that the addition of hypochlorous acid significantly reduced components such as HA and fulvic acid in the MBR effluent. Hu and Shan [58] found that the more organic fractions with a non-polar nature, the more serious fouling was found for both RO and NF. Thus, they examined oxidation with KMnO_4_, aiming to modify the organic polarity in the feed water. It was observed that the polar fractions were significantly increased, especially under 2 ppm dosage. With KMnO_4_ pretreatment, the normalized flux for municipal wastewater effluent in RO and NF systems improved from 0.65 to 0.78 and 0.74 to 0.88, respectively, after 20 h of operation.

Cold-cathode X-ray irradiation is a new technology that decomposes organics and is applied to control organic fouling. Kim et al. [59] evaluated the feasibility of cold-cathode X-ray irradiation to remove O&G in shale gas produced water (SGPW). After dissolved air flotation (DAF) and UF (MWCO: 30 kDa), SGPW (O&G: 3.3 mg/L) was treated under various X-ray irradiation conditions for 6 min. The RO was sustainably operated up to 40% recovery without any flux decline by controlling the concentration of aliphatic hydrocarbon to below 2 mg/L. Their work demonstrated that cold-cathode X-ray irradiation could efficiently remove the aliphatic hydrocarbon.

The H_2_O_2_/UV process has been used in the Groundwater Replenishment System (GWRS) in the United States to remove N-nitrosodimethylamine (NDMA) and 1,4 dioxane from RO permeate [74]. Regarding the effectiveness of removing LMWOCs from RO supply water, it was demonstrated that H_2_O_2_/UV treatment can effectively remove nonionic surfactants, thereby preventing a decrease in flow rate [60]. However, to remove 90% or more of TOC at about 20 mg/L, an H_2_O_2_ concentration of 100 mg/L or more is required. Song et al. [61] investigated H_2_O_2_/UV treatment for removing TOC from groundwater. Preoxidized waters exhibited significantly less decline in NF permeate flux. Longer oxidation times or higher hydrogen peroxide dosages resulted in increased permeate flux. A decrease in specific UV absorbance (SUVA) was observed after H_2_O_2_/UV pretreatment. This SUVA reduction indicates the transformation of humic substances into less- or non-humic substances. It is reported that organic acids, e.g., oxalic acid, acetic acid, malonic acid, and n-butanoic acid, are formed during NOM’s H_2_O_2_/UV treatment [75]. A raw water source spiked with 100 μg/L of alachlor (an herbicide from the chloroacetanilide family) was subjected to H_2_O_2_/UV oxidation (i.e., initial H_2_O_2_ concentration = 1 mM, UV intensity = 1.8 × 10^−6^ einstein/L) for 30 min. No alachlor was detected in the treated water [61].

As shown in Table 3, ozone treatment is the process most often studied to reduce organic fouling. There are two methods: treatment with ozone alone and combined with H_2_O_2_. Pisarenko et al. [70,71,72] evaluated four oxidation processes, UV, UV/H_2_O_2_, ozone, and ozone/H_2_O_2_, for reducing the organic fouling of RO membrane systems for water reuse. UV alone had a minimal impact on organic matter removal and little to no effect on fouling control in flat-sheet tests. UV/H_2_O_2_, ozone, and ozone/H_2_O_2_ effectively decrease total fluorescence and UV254 absorbance for the EEM analysis. In comparison, even at a relatively high UV dose, total fluorescence and UV254 absorbance did not decrease significantly for UV treatment alone. Although a significant decrease in UV254 was observed in the UV/H_2_O_2_ treatment of secondary sewage effluent, the specific flux of RO decreased by 31% after one hour (66% in untreated water). In a comparative test of ozone and ozone/ H_2_O_2_ using MBR-treated water, the decrease in RO specific flux was 7% for ozone alone at a concentration of 3 mg/L but 52% for ozone/H_2_O_2_. Thus, for MBR effluent, ozone alone appears to be the best method for minimizing the organic fouling of RO membranes [71].

Williams et al. [62] demonstrated how ozonation affects membrane fouling when treating chlorophenols. At low feed pH, trichlorophenol (TCP) is not ionized and interacts strongly with RO/NF membranes; the adsorbed TCP displaces water in the membrane pores, causing a significant drop in water flux. Feed pre-ozonation was shown to significantly improve flux and rejection characteristics due to the formation of ionizable organic acid intermediates during the ozonation that do not interact as strongly with the membrane. It was disclosed that the ozone treatment of DOM in surface water, similar to H_2_O_2_/UV treatment, can convert the organic matter into carboxylic acids, aldehydes, and carbonyls, and thus into ionic (hydrophilic) organic matter, thereby preventing membrane fouling [63].

An optimum ozone dosage of 1.2–2 mg/L of O_3_ concentration (0.3 mg O_3_/mg TOC) appears to be sufficient to prevent organic fouling in sewage MBR effluent [64]. Without ozone treatment, the flow rate reduction in RO was about 10–15%, whereas with ozone treatment, the reduction was limited to about 5%. Comparable results were obtained for effluent from a sequencing batch reactor membrane bioreactor process, where a modest specific ozone dose (0.2 mg O_3_/mg DOC at 5.7 mg/L DOC) significantly mitigated fouling on the NF membrane (NF90) compared to filtration without pre-ozonation [65]. Notably, doubling the ozone dose to 0.4 mg O_3_/mg DOC did not provide a significant additional benefit. The EEM analysis supported the effect of ozone dosage in these ranges. Pre-ozonation led to a reduction in total fluorescence, indicating a decrease in the hydrophobicity of the organic matter present in the feed water. Szczuka et al. [66] reported that ozonation (0.8 mg O_3_/mg DOC) minimized RO flux decline by rendering DOC more hydrophilic.

The effectiveness of ozone treatment is affected by reaction conditions. Membrane foulants can be efficiently decomposed and removed with a small amount of ozone by performing treatment in the pH 8 to 10 alkaline range, where the reactivity of ozone and hydroxyl radicals is high [68]. Ma et al. [76] indicated that the degradation of nitrobenzene by ozone exhibited better efficiency at high pH. When pH increased from 8.3 to 10.3, the degradation efficiency increased from 30% to 86% due to the OH ions initiating radical chain reactions to form hydroxyl radicals, which have high reaction reactivity towards nitrobenzene. Sarp et al. [73] examined MBR effluent oxidation with ozone/peroxide in high pH conditions in order to decrease NF membrane fouling. The flux decline was reduced from 29% to 15% when 6 mg/L O_3_ and H_2_O_2_ (1/1 O_3_/H_2_O_2_ mol ratio and pH5.6) were used. A further decrease in flux decline was observed when pH was adjusted to 9.0 (29% to 9%).

Since the extent of LMWOC fouling is mitigated under alkaline conditions, it is feasible to feed the ozone-treated water directly to the RO without neutralization. However, installing AC to remove residual ozone and harmful organics that could not be fully removed by ozonation is preferable [42].

In AOP, membrane fouling can be prevented by changing organic matter into carboxylic acids or ionic (hydrophilic) organic matter, so there is no need to decompose all of the target organic matter. Arai and Hitotsuyanagi [69] proposed measuring the UV absorbance of the feed water and regulating the oxidant dosage based on the measured value. As an example, they showed that stable operation is possible by keeping UV absorbance at 260 nm (E260) below 0.2 after ozonation.

### 2.4. Antifoulnants or Dispersants

It was reported that EfOMs from the activated sludge process treating municipal wastewater lowered the fouling potential caused by other organic foulants like reactive dye and surfactants [77]. One possible mechanism is the formation of the aggregates between EfOMs and nonionic surfactant or reactive dye, which reduces RO fouling. It is, therefore, reasonable to consider whether a specific compound that acts similarly to EfOM could be used as an effective antifoulant.

However, organic fouling (alginate and bovine serum albumin) significantly increased in the presence of commonly used antifoulants, such as polyacrylate or carboxylated dendrimeric-based compounds, when used to condition the membrane surface [78]. These results were derived for macromolecular organic matter, so the effect on LMWOC is unknown. Considering that antifoulants were commonly added in many RO/NF plants where LMWOC fouling has been observed, the antifoulant effect on LMWOC fouling is thought to be limited.

In recent years, antifoulants have been reported to reduce the effects of HA and harmful calcium ions contained in NOM. Yang et al. [79] investigated an environmentally benign antifoulant, polyaspartic acid (PASP). In the presence of PASP, HA fouling decreased even with increasing Ca ion concentration. For other types of HA fouling inhibitors, the following compounds have been identified:

- Polymer compounds having a carbonyl group and a structure including a nitrogen atom bonded to a carbonyl carbon atom, such as PVP and polyacrylamide [80];
- Chemicals including organic amines having two or more nitrogen atoms and five or more carboxyl groups or four or more phosphate groups, e.g., ethylene diamine tetra (methylene phosphonic acid) [81];
- Polymer compounds having a carboxyl group and a sulfonic acid group [82].

To date, no effective commercial antifoulants have been developed for LMWOC fouling, although some promising reports exist. Kronmiller [83] reported the development of a new chemical that can increase the RO flux in stressed systems, like organic oils and organic acids, typically found in the food process and effluent streams. The flux enhancement products were evaluated using a type AFC 99 tubular PA semipermeable membrane. The process stream contained caffeine- and process-derivative organic acids and oils. The test results demonstrated an improvement in flow rate stability compared to a similar run conducted without the antifoulant. A good result was also observed at a wood treatment plant where the stock is a mix of stormwater runoff, creek water, and effluent from a wood treatment plant that manufactures utility poles. The foulants range from rosins and diesel fuel to pentachlorophenol and inorganic foulants. The flux enhancement product was added at a concentration of 100 ppm, along with an antiscalant to control the formation of inorganic scale. The mobile system has been running successfully for 2 months. Kronmiller and Netwig [84] disclosed a process for improving the separation rate of water from a stream of aqueous caffeine and coffee bean extract by RO by adding PVP. So far, the effect of PVP is not apparent. However, it is said that PVP stands far above any other synthetic macromolecules except modified synthetic ones in binding and displays a strong binding affinity toward dissolved small molecules, such as various azo dyes and many kinds of aromatic compounds [85]. Hydrophobic interaction was suggested to play an important role in the binding process [85,86,87]. Fujii and Kawakatsu [80] speculate that the bonding between PVP and polyphenol is due to the interaction between carbonyl groups of PVP and phenolic hydroxy groups via hydrogen bonds.

Shimpo et al. [88] reported a new antifoulant that prevents RO fouling by organic matter mainly composed of humic substances. Although the antifoulant’s primary target is HA, the antifoulant inhibited fouling for secondary effluent (EfOM) to an extent not produced by conventional agents.

So far, no systematic research has been conducted in this field. Thus, it will be necessary to elucidate the fouling prevention mechanism to develop new antifoulants and improve performance.

## 3. Detection and Prediction of LMWOC Fouling and Identification of Foulants

As a troubleshooting effort for the initial flow loss, the presence of LMWOC fouling has been indirectly proved by treating the feed water with an AC filter and confirming that the extent of fouling was reduced [23,24]. In another case, the source of foulants was identified by analyzing various waters within the system using a small RO element (a household 1.8-inch element) [24]. This section will introduce several reported cases that can be applied to the detection and prediction of fouling. Methods for identifying the fouling substances within fouled membranes will also be discussed.

### 3.1. Quick Prediction of LMWOC Fouling Potential

**Figure 2:**
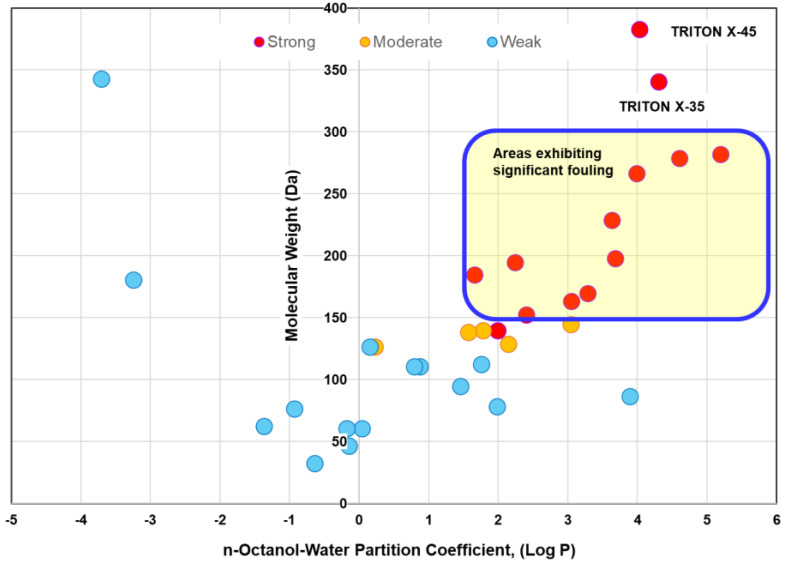
RO fouling potential estimation using an MW–logP matrix chart.

As mentioned in Part 1 [8], two factors are critical for the entrapment of organic compounds within membrane pores: molecule size and membrane–organic interactions. To effectively fill membrane pores, organic molecules should possess an appropriate size. Additionally, their hydrophobicity should enhance their sorption into PA RO/NF membrane matrices [16]. Hydrophobic interaction can be assessed using the n-octanol/water partition coefficients [89]. Organic molecules with high log P values (i.e., high hydrophobicity) tend to have a greater influence on water flux. These findings suggest we might predict the fouling potential from the MW and log P values for known organic substances in the feed water. To verify this concept, two factors for the reported foulants and nonfoulants are summarized in Table 4 and Figure 2 using data from aromatic polyamide (APA) TFC RO/NF membranes. The foulants are assigned into three types: strong, moderate, and weak.

The extent of fouling depends on the membrane materials; operating conditions such as flux and feed flow rate; and concentrations. Thus, the foulants are classified qualitatively in Table 4.

Nevertheless, a clear difference can be seen between the strong and non-fouling compounds. Figure 2 shows that an organic compound that exhibits strong fouling propensities has a log P value of more than 1.5 and an MW ranging from 150 to 300 Da (highlighted by the blue line). In Part 1, fouling is reported as more pronounced when the MWs of organics are slightly smaller than the MWCO. However, actual fouling seems to occur even with larger organics than the MWCO. This may be because fouling occurs preferentially in large pores such as aggregate pores. It should be noted that the foulants of NF membranes might be extended to wider MW ranges depending on the MWCO of NF membranes, as seen in the case of tannic acid [79]. However, this simplified prediction method may not be applicable to foulants based on electrostatic interactions, such as those involving cationic surfactants and amine compounds. Further research is needed to validate its reliability.

As for nonionic surfactants composed of alkyl ethoxylates and alkylphenol ethoxylates, the extent of fouling is affected by both the hydrophobic group and the number of ethoxylate units. Shorter ethoxylate nonionic surfactants are more hydrophobic and exhibit a strong fouling effect, such as TRITON X-35 and X-45. However, it should be noted that longer ethoxylate surfactants with larger MW and lower log P also exhibit a high propensity for fouling [101,102]. Kawakatsu [103] found that higher MW polyethylene glycols (PEGs) (PEG1470 and PEG7100) significantly reduced flux at lower concentrations.

In addition, since long-chain aliphatic hydrocarbons and higher fatty acids have lower solubility, caution is required due to a risk of free oil formation or precipitation during operation as the recovery rate increases [59].

### 3.2. Detection and Prediction of LMWOC Fouling

As fouling with LMWOCs leads to a rapid decrease in flow during the initial stages of operation, the extent of fouling can be assessed relatively easily and quickly using a small module. The testing approach should align with the project development phases:

- Preliminary evaluation of raw water: Bench-scale tests (tests with small elements);
- Preliminary evaluation of pretreatment processes: Bench-scale tests (a flat sheet membrane test might be acceptable for screening purposes);
- The final test of pretreatment: Pilot tests (4-inch or 8-inch elements);
- Detection of fouling during plant operation: Canary tests (small elements).

Selecting an appropriate pretreatment method according to the raw water properties is essential to reduce the membrane’s organic fouling. For this purpose, lab and bench tests can be utilized. For example, when investigating the effects of various combinations of chloramine, ozone, and biological activated carbon applied as pretreatment to reduce the organic fouling of RO membranes and the generation of DBPs, it was found that lab tests lasting approximately 25 h helped confirm the effectiveness of ozone treatment in minimizing the decline in RO flux [66]. Bellona [104] reported that bench-scale tests effectively estimated the potential for the EfOM fouling of the investigated membrane. This was supported by comparisons between bench-scale fouling results and initial operational data obtained from pilot and full-scale tests.

Small membrane devices for organic fouling detection within an operating plant make it possible to detect whether components clogging the RO membrane exist. Further, by monitoring changes in flow rate, pressure, and salinity over time, necessary measures can be taken if these changes are greater than expected [40,105]. The Orange County Water District (OCWD) has been conducting pilot tests of various 8-inch elements when introducing new RO membranes to the GWRS. As a result, valuable data on initial fouling behavior was accumulated [106,107].

SDI and modified fouling index (MFI) are used to predict colloidal and silt fouling, and SDI listed in the ASTM Standards (D4189) has been widely used in RO desalination fields. In general, SDI should be five or less and three or less for large plants. However, no established method or index currently exists to estimate the water quality conducive to organic fouling, including LMWOC fouling. Regarding the question of whether SDI can be applied to organic fouling, it was shown that feed solution chemistry (i.e., pH, ionic strength, and hardness) affected the SDI values of organic-rich feed water to some degree [108]. However, since the NOM fouling of RO membranes is greatly affected by membrane properties such as charge and organic properties, it was suggested that SDI measurements cannot accurately evaluate the true fouling potential by organic matter [108].

Ando et al. [109] reported that even if seawater was pretreated with UF, the SDI value can be high depending on the characteristics of the seawater. They also found that among two types of SDI filters with the same 0.45 µm diameter pore size, the hydrophilic surface-modified MF membrane always showed lower SDI values than the standard Millipore MF filter. In addition, the hydrophobic polytetrafluoroethylene (PTFE) membrane showed the highest SDI values. Normal seawaters do not show this kind of difference, but eutrophic seawaters containing high levels of organic compounds in a closed bay or the Gulf area often indicate unexpectedly high levels of SDI values with very low turbid seawaters. They proposed that the unexpected rise in SDI in the absence of turbidity may be due to the accumulation of hydrophobic organic compounds in the porous structure within the SDI filter. Depending on the concentration of organic compounds in the UF filtrate, a hydrophobic organic compound layer is built on a porous structure inside the SDI filter and blocks the flow channel, causing a rapid decline in flux.

Regarding the influence of membrane type, the SDI value of the MF filtrate of a secondary effluent was in the range of 3.2 to 4.1 for CA membranes from two different manufacturers [110]. In contrast, the average SDI value was in the range of 1.8 to 1.9 for nylon membranes. Handley and Owens [111] compared various membranes, including the ASTM standard membrane (mixed cellulose acetate and cellulose nitrate). They reported that the SDI value varied greatly depending on the membrane type and the properties of the supplied water.

However, when testing sample water containing turbidity components, no significant change in SDI value (SDI > 6) was observed depending on the membrane type [112]. Although not the SDI test, Jönsson and Jönsson [113] reported that in a filtration experiment using octanoic acid as a model foulant, various UF membranes such as regenerated cellulose, PSF, PES, PVDF, and PA were evaluated. Among those, the PA membrane showed the most significant decrease in flow rate. Based on the above results, it might be possible to use hydrophobic membranes such as aramid PA or PES for SDI filters as an indicator of organic fouling and hydrophilic membranes such as nylon membranes as an indicator of colloid and silt components. Arai et al. [25,114] proposed a new method and index applicable to organic fouling. The sample water is first prefiltered with a cartridge filter with a pore size of 0.2 μm to prevent the formation of cake layers on the membrane filter surface and then is filtered under reduced pressure at 500 mmHg using a PA membrane filter with a pore size of 0.45 μm. They measured the time required for filtering every 0.5 L as T1 to T6 and evaluated the filterability from the change in MFFi (=Ti/T1, i = 1 to 6). The MFFi value of the coagulated filtrate from a machine factory wastewater reclamation and reuse facility increased when PA-MF was used but did not increase when CA-MF was used. When the same sample was treated with activated carbon adsorption and the MFFi value was measured again, the MFFi value hardly increased in either the PA-MF or CA-MF cases. When pretreating test water with a cartridge filter, it is preferable to use a CA membrane to minimize the adsorption of LMWOCs.

Other indicators that could be considered include monitoring SUVA and UV, focusing on the hydrophobicity of aromatic compounds. Arai et al. [69] propose monitoring the UV of the raw water to control the amount of UV light irradiated optimally and the amount of ozone or other oxidizing agents added when decomposing and removing harmful organic matter from the RO membrane using AOP. They disclose that fouling can be prevented by controlling the oxidation conditions so that E260 is below a certain set value (e.g., <0.2).

### 3.3. Identification of Foulants

When a fouling problem occurs due to LMWOCs, even if the membrane is disassembled and the surface is observed, no particularly noticeable foulants may be found [115]. Also, one membrane service company stated that the most troublesome case is the foulant that cannot be seen and cannot be sampled [116,117]. The adsorption of LMWOC to the membrane surface is minimal and difficult to detect by standard surface analysis. Tasaka et al. [118] reported that problematic foulants could not be detected by surface FT-IR analysis. Instead, they applied an extraction method with hexane from the hydrochloric acid solution used to clean the membrane, and they succeeded in detecting nonionic surfactants by FT-IR. Attempts have also been made to measure the zeta potential before and after fouling and estimate what substances are adsorbed from the change, but foulants could not be identified in this method [119,120].

Therefore, GC-MS is generally used to identify foulants. The reported methods include extracting foulants from the membrane and directly analyzing them using pyrolysis. Extraction methods include extracting foulants from cleaning solutions with hexane, etc., and extracting them from the contaminated membrane with solvent. In one case, foulants were extracted by methanol (MeOH) since cleaning the fouled membrane with MeOH restored membrane performance [23]. DnBP leached from a reinforced polyester pipe was detected using methylene chloride as an extraction solvent [96]. Martínez et al. [121] developed a comprehensive protocol for extraction using five types of solvents (MeOH, isopropanol (IPA), hexane, dichloromethane, and acetonitrile). In other cases, phthalates were extracted by immersing the membrane in aqueous NaOH, adsorbing it on XAD resin, and eluting it with dichloromethane [122]. Since LMWOC fouling primarily occurs inside the membrane, it may be preferable to gently hand-wash the membrane surface before extraction to remove foulants on the surface. A new method has been reported in which foulants on the membrane surface, rather than the membrane itself, are collected and analyzed using the Headspace Solid Phase Microextraction (HS-SPME) method [121].

The methods for analyzing organic matter in feed water include solvent- and solid-phase extractions. Examples of using Sep-Pak, granular activated carbon, and synthetic adsorbents as extraction materials have been reported [23,26]. It has also been disclosed that organic foulants can be selectively captured by using an adsorbent with the same composition as the PA membrane [37,123]. HS-SPME can also be applied for water analysis [124].

Khan et al. [125] used pyrolysis GC-MS to detect the LMWOCs and pyrolysis products of foulants in seawater desalination RO membranes. Although this study analyzes foulants on the membrane surface, it is also possible to identify LMWOCs in the membrane by removing the accumulated matter on the membrane surface by cleaning it with running water and then removing the nonwoven fabric for the pyrolysis GC-MS. In the case of solvent extraction, it has been revealed that the amount and type of organic matter detected by GC-MS varies greatly depending on the extraction solvents. In particular, hexane provided more information on organic contamination than other solvents (methanol, IPA, dichloromethane, and acetonitrile) [121]. An analysis of ultra-low pressure (ULP) RO membranes applied to secondary sewage effluent detected many GC peaks. Some of them were commonly found in both the fouling layer and the influent water, suggesting they may contribute to organic fouling and, consequently, a decline in membrane flux.

In analyzing organic substances in RO feedwater, a secondary effluent from a petrochemical plant, the SPME technique was employed prior to GC-MS analysis. Aromatic compounds such as phenol, methylphenol, benzeneacetic acid, DnBP, and xylenol were detected [126]. Since DnBP is known to be a harmful foulant, it is presumed to be one of the substances causing the flow rate reduction. Martínez et al. [124] used SPME fibers of four different polarities to measure volatile and semi-volatile organic substances from the RO influent in water reuse applications. They compared the results with those obtained by conventional liquid–liquid extraction. The GC-MS chromatograms varied significantly depending on the SPME fiber used. Polyacrylate and Divinylbenzene/Carboxen/Polydimethylsiloxane (DVB/CAR/PDMS) are complementary fibers that can be used together to characterize organic compounds in influent water. Furthermore, HS-SPME found more compound families than liquid–liquid extraction, making it a powerful method for characterizing organic compounds in influent water. They recommend that further experiments, such as flat-sheet studies, are needed to understand and confirm whether these families of compounds may be potent organic contaminants of RO membranes.

## 4. Cleaning of LMWOCs-Fouled RO/NF Membranes

Guidelines for cleaning RO/NF membranes are provided by membrane manufacturers [11,127,128,129], and many publications also provide information on cleaning agents and cleaning methods [13,130]. Membrane manufacturers recommend alkaline cleaning agents to address organic fouling. However, if inorganic components are mixed with the organic fouling, or if the organic matter has a complex composition, the cleaning effectiveness may be enhanced by adding an anionic surfactant, such as sodium dodecyl sulfate (SDS); a detergent builder, such as sodium tripolyphosphate (STPP) or trisodium phosphate (TSP); or a chelating agent, such as the tetrasodium salt of ethylenediaminetetraacetic acid (Na4EDTA) [99]. The temperature of the cleaning solution is a critical factor, as higher temperatures promote the removal of organic matter from the membrane surface. Because RO and NF plants encounter a wide variety of complex foulants, a single cleaning agent is typically insufficient. Therefore, alternating cleaning agents, such as alkaline and acid solutions, are crucial for sequentially and completely removing these diverse foulants [131,132,133,134].

Meanwhile, regular alkaline and acid cleaning often proves ineffective against LMWOC fouling [23,135,136,137]. In alkaline cleaning, the higher the pH, the higher the flow rate recovery, but the flow rate cannot be completely restored to the original flow rate [115]. Therefore, special cleaning chemicals and methods are required. In this section, cleaning-in-place (CIP) procedures that are considered to be effective for LMWOC fouling are outlined.

### 4.1. Special Cleaning Agents for LMWOCs Fouling

Table 5 shows cleaning agents and methods reported to be effective for LMWOC fouling. Cleaning agents can be divided into alkaline, organic solvent-based, and oxidizing agent-based cleaners. This process typically requires either membrane swelling or organic oxidation to effectively remove organics sorbed within the membrane matrix.

Pressurized filtration cleaning was proposed to replace conventional low-pressure flushing for alkaline CIP [138]. Intentionally passing alkaline solution through the membrane makes it possible to flush out and remove organic foulants trapped inside the membrane. In RO membranes contaminated with wastewater containing nonionic surfactants, the performance was almost restored with 4 to 5 h of pressure cleaning (1.2 MPa) for mild fouling. About 95% of the performance was restored with 18 h of cleaning for severely contaminated membranes. Other alkaline cleaners are effective when they contain a few percent or less of an amide compound, such as nicotinamide [139,160]. When the cleaner’s effectiveness decreases, it is helpful to occasionally use an alkaline cleaner that does not contain an amide compound but a reducing agent or a chelating agent to maintain its effectiveness [140,141].

For organic solvent-based cleaning agents, lower alcohols (MeOH and IPA), butyl cellosolve, and polyols such as EG and PG have been reported, as shown in Table 1. Lower alcohols are also known as rewetting agents for PA TFC membranes [11,161], but they are also effective for cleaning LMWOC fouling. A 30% MeOH solution was applied to clean the elements fouled by phthalates [23]. Considering the safety of handling MeOH, cleaning was performed off-site.

Butyl cellosolve is reported to effectively remove foulants attached to UF membranes treated with electrodeposition paints containing liquid [162,163]. The following example may be the first reported instance of RO CIP using butyl cellosolve solution at a steel pipe manufacturing plant where a sudden drop in flow rate was encountered due to the leakage of water–glycol-based hydraulic fluid. The problem was that regular acid and alkali cleaning was ineffective in removing membrane contamination caused by hydraulic fluid, and the number of cleanings had to be increased. After consulting with the membrane manufacturer, special cleaning using 10% butyl cellosolve was performed, which removed membrane contamination caused by hydraulic fluid and restored the flow rates [142,143]. The effectiveness of 50% MeOH or 10% butyl cellosolve solutions in removing fouling from RO membranes used to treat rolling mill wastewater in the steel industry was also confirmed [147].

Kawakatsu et al. [103] investigated the mechanism of fouling caused by nonionic surfactants and PEGs, and explored effective cleaning methods. Alkaline solutions (pH 12 NaOH, 18 h) and 30% MeOH were ineffective in recovering membranes fouled with PEGs. On the other hand, a mixed solution of polyol and alcohol (polyol: 70% EG; solvent: 30% MeOH) restored the membrane flux to 80% of the original. It is presumed that the membrane skin layer was swollen, and PEG molecules were removed by EG molecules with similar chemical properties. The effectiveness of a mixed solution of polyol and alcohol (polyol: 70% EG or PG; alcohol: 30% MeOH or ethanol) has also been confirmed for removing nonionic surfactants [144,145].

Several chemicals containing oxidizing agent-based cleaning agents have been proposed, namely, high-concentration nitric acid, high alkaline solutions containing hypochlorite, hydrogen peroxide, dichloroisocyanurate (DCC), etc. In addition, membrane chemical suppliers have their own proprietary oxidizing agent-based cleaning technology and have published their effects [136].

Maki et al. [164] investigated the cleaning of organic-fouled UF membranes. When UF modules utilized in the polishing loop of ultrapure water (UPW) systems became severely contaminated by ion exchange resin leachate, cleaning with high-concentration nitric acid proved effective. So, cleaning tests were conducted for fouled RO membranes used in the pure water treatment systems [118]. Flux recovery with nitric acid was superior to acid and alkaline cleaning. However, they mentioned that caustic soda with a pH of 11 should be selected for RO membrane elements because some materials for spiral-wound elements cannot withstand high-concentration nitric acid. It was also disclosed that compared to nitric acid cleaning alone, the three-step cleaning is more effective: firstly clean with an aqueous solution of NaOH with a pH of 12 or higher, followed by nitric acid with a pH of 2 or lower, and then an alkaline (pH 12) solution [149]. In addition, a mixed solution of polyols such as EG and nitric acid has a synergistic effect that significantly improves the cleaning effect [150]. Nitric acid cleaning was applied in an RO plant producing process water for a pulp mill built in 2004 [148]. The RO membrane started with a higher flow rate than initially predicted. However, the standard cleaning procedure was ineffective, so the flow rate progressively decreased in the first six months. In this case, most of the flow rate was restored when the membrane was cleaned with 7% nitric acid. However, nitric acid is highly corrosive and poses significant risks to operators and equipment, particularly membranes. Therefore, specialized cleaning agents were subsequently developed.

Finally, highly alkaline cleaners containing hypochlorite or hydrogen peroxide might be the best method to perform on-site. The cleaning of semipermeable membranes fouled by organic substances with cleaners containing oxidizing substances (hydrogen peroxide or hypochlorite) at a pH of 9 or higher has been disclosed, but no examples of RO membranes were shown [151]. In addition, Moch et al. [165,166] have reported a new cleaning procedure using a high pH NaOCl solution for removing biological contaminants from RO modules. In this cleaning procedure, 250 mg/L of NaOCl with a high pH (11.8–12.0) is contacted for about 30 min and circulated for 15 min. However, the removal of LMWOCs was not mentioned. Freeman et al. [152,154] conducted cleaning tests using high pH hypochlorite at a semiconductor factory wastewater reuse RO plant installed in Chandler, Arizona, because regular cleaning was not satisfactory. First, acid cleaning was performed, followed by alkaline cleaning for 30 to 60 min. Then, NaOCl solution was added to adjust the chlorine concentration to 50 to 80 mg/L, and circulating cleaning was tested for about 6 h. They concluded that since hypochlorite is not a strong oxidizing agent at high pH, careful application seems to extend membrane life. However, they also mentioned that the membrane may be accidentally damaged during cleaning (e.g., if exposed to hypochlorite at neutral pH) and that cleaning operations must be performed according to the established procedures.

In cleaning membranes operated for half a year at an actual plant, a decrease in salt rejection was observed when cleaning with a chlorine concentration exceeding 50 ppm, so a cleaning method with a chlorine concentration of 10 ppm or less has been proposed [156]. Since heavy metals may have accumulated on the surface of a fouled membrane, it is preferable to remove heavy metals by acid cleaning before performing oxidative cleaning, as seen in the tests conducted in Chandler.

Farooque et al. [153] reported that standard acid and caustic cleaners were ineffective for cleaning NF membranes used in a seawater application. However, a more pronounced cleaning effect was obtained when hydrogen peroxide (1–2.5%) was added to a high-pH hypochlorite solution. They also reported that based on a test using a 4-inch element, a chemical cleaning procedure using NaOCl/H_2_O_2_ at high pH was very effective in removing fouling from the NF membrane surface without damaging the membrane and restoring membrane performance. However, attention should be paid to the effect of alkaline hypochlorite on RO/NF membranes. Since flow rates and salt rejections increase when new membranes are treated with high pH hypochlorite [167,168,169,170], it remains to be seen whether the flow rate recovery during cleaning is due to organic removal or an effect on the membrane structure. Other examples of high-pH oxidants include chloramine compounds (e.g., monochlorosulfamic acid) [159].

DCC has also been reported as another oxidative cleaning component [137]. Conventional cleaning methods were ineffective when a boiler water RO plant in Schkopau, Germany, experienced a sudden drop in flow rate shortly after starting operation. The flow rate could be restored by using DCC. This oxidative cleaning was applied three times but was subsequently discontinued due to an obvious negative effect on salt rejection. However, a subsequent study showed that the normalized salt rejection of the DCC-exposed membrane remained stable at 99% without sacrificing flux [171]. In contrast, the salt rejection of the chlorine-exposed membrane dropped to 80%. Since there has been no detailed study of DCC for LMWOC foulant removal, further studies will be required, including the effect of pH and concentration.

The following case is a pilot trial for a reuse system consisting of an MBR-RO in a textile dyeing facility [172]. The permeability of the RO membrane dropped significantly after a very short run time. An autopsy of the membrane revealed that the membrane was fouled with a very thin layer of tightly adhered organic dye. Oxidative cleaning with a chloramine-based cleaner and chlorine dioxide solution was attempted, but the permeation flux did not improve. However, when a high pH cleaning agent was applied as a secondary step after cleaning with chlorine dioxide solution at pH 5.7, the fouling was completely removed, and the membrane performance was restored without affecting the salt rejection of the membrane. Although chlorine dioxide has no direct effect as a cleaning agent, it was found to change the chemical properties of organic dyes, making them more soluble in high-pH cleaning solutions.

### 4.2. Timing of Cleaning

The following guidelines are provided by membrane manufacturers for the timing of membrane cleaning [11,127]:

- Normalized permeate flow has decreased by 10% since startup or last cleaning.
- Normalized salt passage has increased by 10% since startup or last cleaning.
- Normalized pressure drop from feed to concentrate has increased by 15% since startup or last cleaning.

Since a significant drop in flow rate is often associated with LMWOC fouling, frequent cleaning is required according to this guideline. However, specialized cleaning agents may only be required in limited cases, such as the following:

- The concentration of leachables from plant construction materials decreases over time to a level that does not interfere with the RO operation.
- Measures are taken to prevent problematic substances from entering the plant, such as fouling caused by hydraulic fluid leaks and installing a GAC filter.

**Figure 3:**
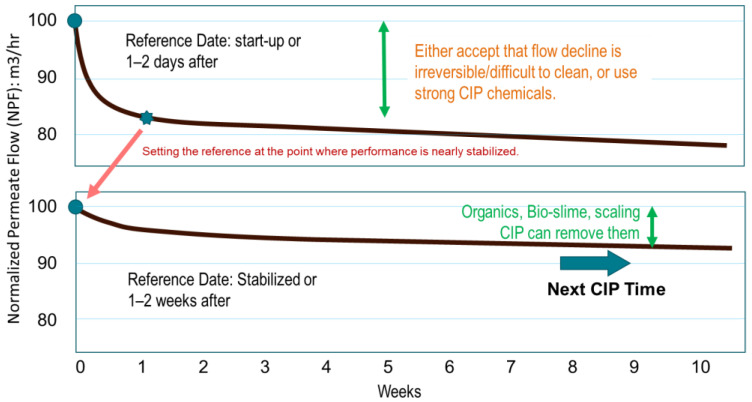
Timing of cleaning for LMWOC-fouled RO/NF membranes.

On the other hand, if foulants remain mixed in the feed water, the flow rate will quickly decrease even after rigorous cleaning. This necessitates frequent cleaning, leading to increased operation and maintenance (O&M) costs. Therefore, as shown in Figure 3, instead of cleaning when the flow rate decreases by 10% from startup, a cleaning method based on the point when performance stabilizes should be suitable for municipal and industrial wastewater reuse plants [115,173]. This cleaning method has been implemented in the GWRS in OCWD [174]. By shifting the reference point, the frequency of required cleaning can be reduced. In other words, this O&M idea is to accept LMWOC fouling as irreversible and focus on removing other particulate matter and biofouling that accumulate on the membrane surface by regular cleaning. This approach should also eliminate the need for specialized cleaners designed for LMWOC fouling.

## 5. Operational Methods

Although a rapid flow rate drop is observed immediately after starting operation due to LMWOC fouling, it usually reaches a steady value within 1–2 weeks of operation. If a flow rate drop is known in advance through pilot tests, etc., Carney [175] advises that much higher fouling allowances (such as 30–50%) should be taken into consideration such that the pump pressures are significantly higher than design projections. Meanwhile, if fouling is not predicted, responding by applying frequent CIP or installing AC will be necessary. A practical and simple alternative response is to accept the irreversible fouling and continue the operation as usual.

Sehn [137] explained the fouling phenomena and corrective measures experienced at a power station for boiler feed water treatment. The flow had decreased dramatically during the first month of operation due to aliphatic halogenated hydrocarbons. Conventional cleaning methods have not been effective, but with chloroisocyanurate, the flow was able to be recovered. A similar flux decline was observed when the new third train was started. In this case, the oxidative CIP was not applied. The new train continued running operations with cleaning at pH 13, although the effect of this CIP was limited. The permeate’s high and stable quality, especially the low silica concentration of typically 5 ppb (from a feed concentration of 1 to 2 ppm), was considered more beneficial than a further reduction in the feed pressure (about 4 bar). The same O&M procedure was reported for wastewater RO treatment in a steel plant where the feed had high COD and TOC [176].

EfOM is present in the RO feed water in municipal wastewater reuse plants, causing a significant initial flow decline. In response, many plants have now adopted the approach of accepting irreversible fouling and continuing operation as is [115,174,177,178,179,180,181]. The West Basin Municipal Water District had experience with this performance phenomenon and established an operating philosophy that accepts the rapid decline in specific flux [177]. Based on this concept, the RO cleaning strategy was evaluated to minimize energy and CIP costs [174]. Majamaa et al. [178] reported O&M procedures to maintain RO performance at a domestic wastewater reuse plant in the Netherlands. They focused on maintaining stabilized performance and preventing biofouling by utilizing preventative cleanings at a pH of 11 rather than restoring the initial flow.

The above examples illustrate instances where LMWOC fouling is allowed to occur, followed by normal operational practices. The following subsection will review a few examples of reducing fouling by changing the operating pH.

### 5.1. High pH Operations

Operation at a high pH has several advantages and has been applied in various fields [182]. For example, when the silica concentration in the raw water is high, the recovery rate cannot be increased due to a solubility limit. The solubility of silica is increased by raising the pH to 10 or higher, which makes it possible to increase the recovery rate [183]. Other applications include seawater desalination and ultrapure water production processes, where boron rejection, which is low at neutral pH, can be improved by raising the pH to approximately 10 or higher [184,185].

Another benefit noted is the reduction in organic fouling. Hitotsuyanagi et al. [186] developed an RO process for treating organic wastewater by adjusting the feed pH to 10 to 13. They speculate that the working mechanism is the same as that of alkaline cleaning, so this method can effectively prevent the adsorption of organics to the membrane. It is also assumed that both the membrane and any organic substances have a negative charge at pH > 9 [11,187]. For these reasons, high pH operations have been widely adopted for difficult waters such as oily produced water and refinery wastewater [188,189,190].

The following mechanisms are thought to reduce organic fouling at high pH:

- Increase in negative charge of membrane: Increase in hydrophilicity (contact angle) and swelling of membrane;
- Electrostatic interactions: Decrease in sorption of organic compounds;
- Hydrolysis of certain organic compounds: Phthalates;
- Increase in solubility of organics: Hydrocarbons, fatty acids, etc.

The negative charge of PA membranes comes from unreacted carboxyl groups. The degree of dissociation (or pKa) of carboxylic acid groups can vary significantly depending on their immobilization environment: bulk aqueous solution versus surface-immobilized species. It was observed that the degree of dissociation of polyelectrolytes such as polyacrylic acid shifts to the higher pH side compared to acetic acid [191]. In the case of polyethylene film having a high density of carboxylic acid, ionization of these carboxylic acid groups begins at approximately pH 6 and is complete at a pH of around 11. Along with this ionization, the contact angle decreases as the pH increases from 6 to 11 [191]. The same behavior for contact angle changes is observed for the TFC RO membrane (XLE) [192]. The membrane’s charge can also be estimated from zeta potential measurements, which decreases from the isoelectric point at pH 4–4.5 to pH 10. From the above, it is thought that in the alkaline region of pH 10, the dissociation of carboxyl groups progresses completely, and hydrophilicity increases. It is also thought that the membrane swells as the membrane charge increases [193]. Thus, the membrane pore size increases, so it may become difficult for LMWCO to be trapped within the expanded pores.

Since the membrane and organics, such as organic acids and phenolics, are negatively charged at high pH, they repel each other through electrostatic interactions, resulting in reduced fouling. For example, the flow decline for the fatty acid (octanoic acid) is minor at pH 9. [194]. A severe flux decline was observed by adding SDS (0.3 g/L at pH 7) when evaluating wastewater treatment in the textile reactive dyeing process. This decline was prevented when the pH of the wastewater was raised to 10 due to the repulsive electrostatic interactions [195].

It is also thought that some organic substances are hydrolyzed in alkali, and their foulant activity is reduced. For example, phthalic acid esters are hydrolyzed by alkali. Dimethyl phthalate (DMP) is decomposed into the mono- and diacid hydrolysis products. It is reported that monoacid is quickly generated at 30 °C in a 0.01 M NaOH solution [196]. Thus, fouling with phthalates may be minimized under alkaline conditions due to mono- and di-carboxylate formation.

Another favorable effect is the increased solubility of organic substances such as hydrocarbons and fatty acids. The solubility of oil in San Ardo-produced water in California increases from 225 mg/L at pH 7.5 to 320 mg/L at pH 9.5 [197]. Naphthenic acid shows a similar organic solubility increase [193]. Therefore, free oil formation or precipitation can be prevented at high pH, even under higher recovery conditions. The pilot test at the San Ardo field confirmed that RO membrane fouling could be controlled by raising the pH to 10.6–11.0, where the feed soluble oil concentration was 80 mg/L. Even though a rapid flow decline was observed at the beginning (1st stage: ca. 20% and 2nd stage: ca. 30%), there was no further fouling after day 45 in either stage [197]. Franks et al. [188] reported the RO membrane’s long-term performance and the element’s integrity for a produced water treatment plant containing high O&G concentration (38 mg/L) at pH 10.7. After six months of operation, a lead element was taken from the RO system and subjected to a detailed analysis. The retest indicates only a 16% flux loss compared to the original factory wet test performance.

As for higher fatty acid, Brinck et al. [198] studied the adsorption of octanoic acid (sodium octanoate concentration: 5.9 mM) on a planar, hydrophobized silica surface by means of in situ null ellipsometry. A sharp increase in the amount adsorbed was observed when the concentration of undissociated acid approached the saturation concentration as the pH was decreased.

Next, the optimum pH is considered for reducing fouling. The first case is an RO plant treating industrial wastewater consisting of an evaporation condensate containing LMWOCs [199]. The main components are polyol, trimethylolpropane, formic acid, methanol, and formaldehyde. Severe fouling problems were encountered during the first year of operation. The pH of the condensate is 3 and initially not adjusted. However, alkali was added to the condensate by accident, and it was noticed that the membrane performance improved markedly. The influence of pH on flux and fouling was then investigated. It was found that when the pH was below 10, the flux declined rapidly, resulting in a need for frequent cleaning. After this finding, the pH of the feed was controlled to about pH 10.5.

**Figure 4:**
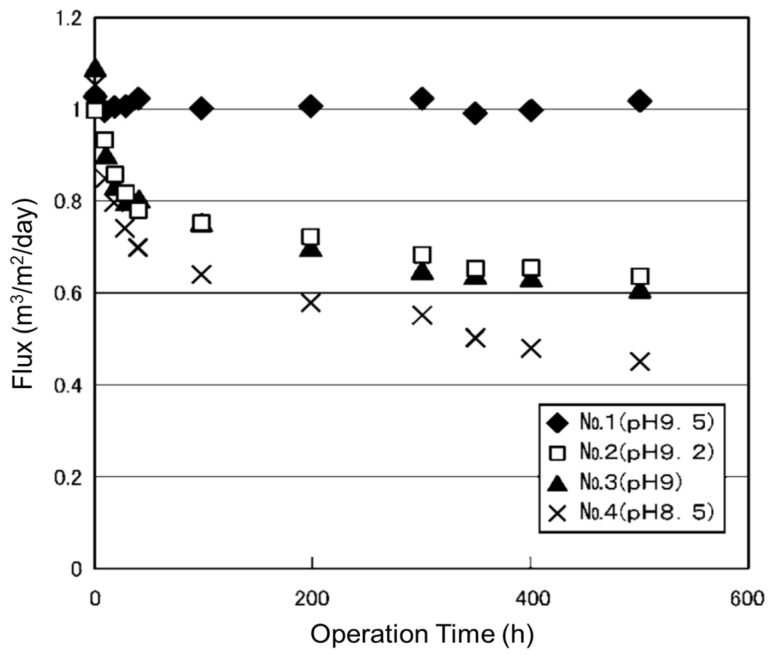
Effect of feed pH for treating wastewater containing nonionic surfactants [200]. (RO membrane: NTR-759HR; pressure: 1.47 MPa; recovery: 90%; feed TOC: 10 mg/L).

The following case concerns the effect of pH when treating wastewater from an electronic device manufacturing plant containing nonionic surfactants with RO [200,201]. NaOH was added to the wastewater (pH 7.2, TOC 10 mg/L) to adjust the pH to 9.5 (No. 1), 9.2 (No. 2), 9.0 (No. 3), or 8.5 (No. 4), and RO filtration was then performed under conditions of a 90% recovery rate. Figure 4 shows the change in flux over time in each RO operating condition. The decrease in flux was suppressed by setting the feed pH to 9.5 or higher. As mentioned in the above two cases, it was found that a slight increase or decrease in pH can drastically change fouling behavior, but the mechanism behind this is unclear. Further research is needed to better understand this phenomenon.

### 5.2. Low pH Operation

TFC PA membranes have amphoteric properties, i.e., negatively charged in neutral and alkaline conditions and positively charged in the acidic range. This property can be considered effective in treating feed water containing cationic LMWOCs, such as cationic surfactants at low pH.

However, there are some points to consider when implementing this idea. First, the membrane’s charge differs between APA and PPA membranes, and the isoelectric point of the membrane varies from 2.8 to 5.0 [202,203]. Although, depending on the report, there is variation in the zeta potentials, the APA membranes typically show a zeta potential of +10 mV or more at pH 3, except those coated with PVA [192,204,205]. Secondly, the positive charge of the membrane is thought to come from amine groups remaining in the membranes [205]. The amine groups can easily be oxidized by oxidizing agents such as chlorine and lose their positive charge [206]. As mentioned above, when treating cationic LMWOCs at low pH, it is crucial to select an appropriate membrane and prevent any contact between the membrane and oxidizing agents.

Childress and Elimelech [207] measured the zeta potential after contacting a test membrane with an anionic surfactant (SDS) and a cationic surfactant (dodecyltrimethylammonium bromide, DTAB). Even when contacted with DTAB below the isoelectric point, the zeta potential was increased compared to the uncontacted membrane. It is speculated that hydrophobic interactions dominate in the lower pH range because the membrane and surfactant molecules are similarly charged. Therefore, it is thought that some degree of surface fouling will occur even at low pH, but the key is whether internal fouling can be prevented. Boussu et al. [208] investigated fouling by surfactants using four types of NF membranes. The zeta potential of the Desal51HL membrane is 3.5 and −12.6 mV at pH 3 and 6, respectively. Although the adsorption of cetrimide to the membrane is high, at 14.0 mmol/m^2^ at pH 6 and 2.7 mmol/m^2^ at pH 3, the relative flux was almost the same, at about 90%. With PPA-based membranes, significantly lowering the pH might not be necessary, and fouling may be reduced even at pH 5–6. Ochi and Chiba [209] disclosed a similar RO/NF process where feed water containing cationic or nonionic surfactants is treated with pH 6 or less. They indicated two examples where SU600 membranes treated feed waters containing cationic/anionic surfactants at pH 5.8 and cationic/anionic/nonionic surfactants at pH 3.9. A higher flow rate could be maintained compared to operation at pH 7. Ikuno and Maeda [210] developed an RO separation process for wastewater containing a cationic surfactant at a pH range of 3 to 5. The examples show data for wastewater containing 2 mg/L of a monoalkyl ammonium chloride cationic surfactant treated with an APA RO membrane (ES-20) at pH 4, 5, 7, and 9. When the pH of the RO feedwater is 7 or 9, the permeation flux decreases significantly over time. In contrast, when the pH of the RO feedwater is 4 or 5, the degree of decrease in the permeation flux is insignificant even after 30 days, and membrane fouling by the cationic surfactant is prevented.

## 6. Low Fouling RO/NF Membranes and In Situ Treatment

PPA-based NF and CA RO membranes have been reported as low-fouling membranes against LMWOCs. For example, it was disclosed that a TFC membrane consisting of a mixture of ethylenediamine and piperazine as the amine component does not show permeate flow decline even when treating a nonionic surfactant [211]. Therefore, applying such membranes to feed water containing LMWOC foulants is considered adequate, but they also have some shortcomings. PPA-based membranes, a type of NF membrane, are generally not suitable for producing high-purity water. CA RO membranes require higher operation pressures than TFC RO and have lower rejection for organics and silica. For example, in municipal wastewater tests, the TOC rejections were reported to be 89% and 99% for CA membranes and TFC RO, respectively [212]. In addition, a significant difference was observed in the rejection of TCP, known as a significant foulant, at 9% and 100% for CA and APA membranes, respectively.

PPA or CA membranes can be used as pretreatment for TFC RO or in a two-pass system. However, as reported by Oda et al. [52], a significant flow rate drop occurs in the second-pass TFC RO when treating wastewater containing LMWCOs, even if CA membranes are used as pretreatment. When the flow rate drop happened in the two-pass system consisting of CA RO/TFC RO, introduced as a case study in Part 1, it was suspected that the flow decline was due to leachables from the first-pass CA RO element. Considering the results of Oda et al. [52], the possibility that the flow loss in the second-pass RO was due to organics contained in the raw water cannot be ruled out.

The development of low-fouling membranes has attracted the attention of researchers, and many studies have been conducted. Some of these studies were previously introduced in the surfactant section of Part 1 [8]. This section will focus on posttreatment, which can be treated on-site or off-site as a corrective measure.

**Figure 5:**
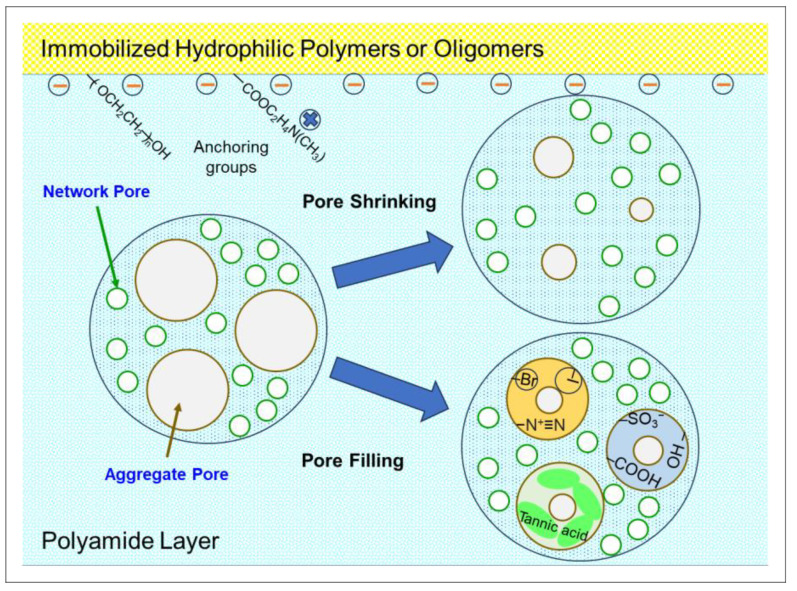
In situ surface and pore modifications to alleviate LMWOC fouling.

There are two possible causes of LMWOC fouling: surface and internal fouling. An overview of corrective measures corresponding to these fouling mechanisms is shown in Figure 5. Coating with hydrophilic polymers or oligomers is an effective method for preventing surface fouling. The important point is the immobilization of the coating layer. To fix the hydrophilic polymer to the membrane surface, it is possible to utilize electrostatic interactions or to introduce pendant groups (polyethoxylate, quaternary ammonium, etc.) that can provide an anchoring effect. Next, pore shrinking or pre-filling is effective in preventing LMWOCs from being trapped in aggregate pores to avoid internal fouling. These will be described in detail in the following subsections.

### 6.1. Membrane Surface Modification

Surface modification is effective in imparting anti-fouling properties to PA RO/NF membranes, and many research works have been carried out on this subject. Coating materials such as PEG-, polydopamine-, and zwitterionic-based ones have been examined depending on the purpose of the modification [213]. When applying hydrophilic polymers, it is crucial to investigate how to immobilize them to the RO membrane surface. Usually, the coating layer is heat-treated, crosslinked, or grafted to be immobilized, but these methods are often unsuitable for the on-site or off-site treatment of RO elements. Insolubilization treatments thought to be adequate for on-site application include the copolymerization of monomers that can be expected to have an anchoring effect, such as PEG and cationic pendant group, or surface treatments that utilize electrostatic interactions.

An example of the use of the anchoring effect is shown for PVA. PVA has been widely used in commercial RO membranes to form a protective layer and impart a low-fouling nature [214]. Heat treatment [215] or crosslinking treatment [216,217,218] is performed to make the PVA layer insoluble during the membrane formation, but this is not suitable for on-site treatment. Hayakawa and Kawakatsu [219] disclosed a method of surface modification using modified PVA with polyethoxylate as the anchoring group. When this membrane was applied to wastewater treatment, the durability of the anti-fouling effect was improved compared to conventional PVA treatment. They also reported a similar treatment that applies this method to betaine compounds [220].

Polycations can be used for immobilization treatment using electrostatic interactions. In the neutral range, PA membranes have a negative charge, so when they come into contact with polycations, the polycations are fixed to the membrane surface. Uemura et al. [221] have disclosed a composite membrane modification using this method. Zhou et al. [222] reported that modified membranes in contact with PEI showed a low fouling propensity against DTAB.

It has been revealed that PECs are formed by contacting a polyelectrolyte membrane with an oppositely charged polyelectrolyte, and this method can be used to immobilize various hydrophilic polyelectrolytes [54,223]. For example, it was reported that carboxymethyl cellulose (CMCL) coating is effective against organic fouling, but heat treatment is required to immobilize it [224]. Therefore, it may be possible to fix the CMCL layer by first fixing the polycation and then contacting it with a CMCL solution.

A method that further advances this idea is LbL assembly or modification [225,226]. In this method, a polycation and a polyanion are alternately deposited on a substrate and are adsorbed by electrostatic interaction. The advantage of this method is that various polycations and polyanions can be chosen. Kurokawa et al. [227] measured the sorption isotherms of water and some organic solvents for various PECs. They observed that the PEC consisting of polystyrene sulfonate (PSS-Na) and an ionene shows a lesser organic sorption nature (methanol, acetone, and benzene).

As mentioned in the previous section, PEC membranes have been reported to have excellent LMWOC removal properties, so the PECs are thought to be effective for surface modification [52]. Ishigami et al. [225] investigated surface modification using the LbL method with PSS-Na and poly(allylamine hydrochloride). Interestingly, 6-layer and 12-layer membranes showed appreciably low fouling against DTAB despite the negative charge on the membrane surface. This phenomenon may suggest that the PEC membrane prevents DTAB from being sorbed to the PEC matrix.

To apply the LbL method in the field, reducing the number of alternating adsorption treatments is desirable. Tanaka and Osawa [228] applied pressure treatment (0.75 MPa) to cation- and anion-modified PVA for 2 and 5 h, respectively. The results showed that a stable PVA layer was formed after two-step treatments.

Examples of other material modification methods, such as PEG and lignin, are introduced here. PEG is chelated and immobilized onto the PA surface by ion–dipole interaction between ether oxygen atoms in the PEG backbone and –COONa in the PA layer [229]. The TFC RO membrane was first immersed in a NaOH aqueous solution (pH 14) for 10 min to convert the –COOH in the PA layer into –COONa. After rinsing with deionized water, the membrane surface was then contacted with a PEG aqueous solution. The PEG-modified membrane showed less fouling characteristics for the DTAB solution. de Jubera et al. [230] reported NF membrane modification with aramide dendrimers with oligoethylene glycol chains on their peripheries.

Biopolymer lignin was deposited onto the surface of RO membranes using a simple filtration method [231]. Alkaline lignin is deposited to the membrane surface via both hydrogen bonding and π-π interaction. Lignin deposition reduces the surface roughness of the membrane and enhances its negative charging. The lignin-deposited membranes were applied to treat real effluents of dyeing and papermaking, and they performed much better than the unmodified membranes.

### 6.2. Pore Size Reduction

Internal fouling is considered more important than surface fouling when encountering fouling caused by LMWOC. When the LMWOC is trapped in aggregate pores or large-sized network pores, water permeation is hindered, and the flow rate decreases. Bartels and Franks [115], in their work addressing membrane structures less susceptible to fouling, suggest that an ideal PA membrane could be made with only micro-cycles (network pores). This configuration could potentially eliminate the rapid organic fouling observed in many wastewater RO plants. For NF membranes specifically, Boussu et al. [232] proposed a slightly different idea that a low volume fraction of small pores (network pores) is desirable if the feed solution contains dissolved uncharged or charged organic components. Fujioka and Nghiem [233] also noted that tighter membranes are less susceptible to flux decline than looser membranes.

In developing such low-fouling membranes, the rejection rates of boron and IPA, which are sensitive to the membrane pore structure, can be used as indicators for tight membranes. When evaluating currently available membranes against the ideal membrane described by Bartels and Franks [115], SWRO membranes fall into this category. It has been reported that SWRO has a higher boron rejection and smaller pore size than BWRO. Therefore, SWRO membranes may be a suitable option to mitigate LMWOC fouling. However, due to its lower permeability compared to BWRO membranes, it has the disadvantage of requiring higher operating pressures. Thus, the SWRO becomes viable only if its pressure requirement is lower than the pressure increase caused by LMWCO fouling in BWRO systems.

To prevent fouling, reducing aggregate pore size is crucial. Pore shrinking and pore filling are possible methods that allow in situ treatment. Pore shrinking is mainly performed by heat treatment. Hot water treatment has been considered to improve stability during high-temperature operations and increase the rejection of LMWOCs and boron [234,235]. Hot water treatment is also used to sanitize membranes in the pharmaceutical and medical fields. About 15–20% flow decline was typically seen after initial hot water sanitization at 80 °C [236]. Eriksson [237] reported that heat disinfection at 90 °C of 4-inch diameter RO elements decreased the water permeability by 25%.

Fujioka et al. [233,238,239] investigated the effects of heat treatment on membrane fouling resistance and the rejection of small and neutral solutes. Boron rejection by the ESPA2 membrane was enhanced by heat treatment (80 °C and 4 h) from 26 to 68% (when evaluated at a permeate flux of 20 LMH) [238]. Positron annihilation lifetime spectroscopy (PALS) revealed that heat treatment did not significantly influence the free-volume hole radius of the membrane. Thus, they suggested that changes in the other membrane properties, such as free-volume fraction and thickness, may be the main causes of improving boron rejection.

To assess the membrane fouling propensity, the filtration of the secondary effluent was conducted at an elevated permeate flux of 30 LMH [233]. The permeate flux of the virgin RO membrane initially dropped by 20% within the first 5 h of filtration and subsequently decreased gradually by about 10%. In contrast, the permeate flux of the heat-treated (70 °C and 24 h) ESPA2 membrane exhibited only about 5% drop within the first 2 h of filtration and a total of approximately 12% decline at the end of the experiment. In this case, fouling caused a considerable permeability reduction from 4.1 to 2.9 LMH/bar for the virgin membrane, while the permeability of the heat-treated membrane revealed a negligible reduction from 2.9 to 2.6 LMH/bar. However, it should be noted that although the relative flux of the original membrane was significantly reduced, the absolute flux is still higher than that of the heat-treated membrane. Therefore, finding suitable heat treatment conditions for each membrane type is imperative.

Pore filling can be performed by immobilizing organic or inorganic substances that react with the amino or carboxyl groups remaining in the PA membrane or by sorbing fillers such as tannic acid to large pores of NF membranes [240]. Inorganic substances include bromine and iodine, but iodine has a large atomic radius, so there is a possibility that the flow rate will decrease significantly. So, bromine treatment may be preferable. In this case, stabilized bromine has been proposed to prevent the oxidative deterioration of the membrane [241]. The treated membranes have an improved boron rejection.

Treatment with compounds that react with residual amines in the PA membrane can enhance the rejection of sulfate ions and IPA [242]. These include a carboxylic acid ester, a carboxylic acid anhydride, an amine-reactive organic halogen compound, an ethylenically unsaturated hydrocarbon, or a 1,3-propanesultone. These pore-filling compounds reduce the pore size, making a decrease in flow rate inevitable. To minimize the reduction in flow rate, introducing hydrophilic sulfonic acid groups, carboxyl groups, or hydroxyl groups may be effective. Treatment with sulfonating agents such as 1,3-propanesultone has been proposed to introduce sulfonic acid groups [242,243]. Another method that seems to be promising for pore filling is nitrous acid treatment or diazotization [244,245]. Cadotte and Schmidt [246] developed a treatment method for treating the PA RO membrane with a nitrous acid solution. Nitrous acid reacts with primary amines to form diazonium salts, which produce azo compounds in azo coupling reactions. The phenolic hydroxyl group can be introduced by heating the diazotized membrane. Methods of adding a compound that reacts with diazonium salts during or after diazotization treatment have been investigated. For example, potassium iodide is added to introduce iodine [247], and sulfamic acid or polyfunctional phenol is added to introduce sulfonic acid or phenolic hydroxyl groups [248,249]. In particular, it was shown that boron rejection is improved by coexisting m-phenylenediamine, a monomer of the APA RO membrane [250]. In this way, diazotization treatment may be able to form new network pores within aggregate pores through azo coupling. In addition, since surface modification can be performed simultaneously, it is considered a noteworthy in situ treatment technology.

## 7. Applications for Enhancing RO/NF Performance and Rejuvenation

Rapid pressure increases due to fouling can hinder steady operation and increase O&M costs. However, LMWOC fouling reduces aggregate and large network pore sizes, improving the rejection of LMW compounds such as IPA, urea, NDMA, and boron. It may also be used as a performance recovery (rejuvenation) agent when the membrane is oxidized or physically damaged. It is thought that LMWOC improves membrane performance by filling micro defects on the membrane surface and large pores in the membrane matrix. Although not an RO membrane application, the author has experienced that when it was difficult to reproduce the membrane performance of asymmetric pervaporation membranes such as poly(parabanic acid) [251] and polyacrylonitrile [252], pouring a few drops of epoxy resin extract to the ethanol or acetic acid feed solutions dramatically improved the separation factor. Since this epoxy resin extract is also a significant foulant in RO/NF, a similar effect can be expected.

Given these potential advantages for RO/NF operations, this section discusses reported applications, categorized into performance enhancers and rejuvenation agents (RJAs).

### 7.1. Application as a Performance Enhancer

There is a continuous demand to improve the performance of RO/NF membranes, e.g., fouling and chlorine resistance, salt rejection, etc. For example, Wilbert [253] examined treating PA RO/NF membranes with a homologous series of polyethylene oxide-based surfactants to enhance fouling resistance. This idea originated from medical research on the prevention of protein adsorption. However, this attempt was unsuccessful due to a significant flow decline by nonionic surfactants.

Improving rejection is the most critical requirement for specific components and applications. TFC PA RO/NF membranes are highly effective at removing ionized inorganic and organic substances, but the rejection of uncharged LMW substances is not high enough. Examples of LMW organic and inorganic substances that are problematic in RO treatment are shown in Table 6.

In the production of UPW for use in the microelectronics industry, the primary role of RO is to remove ionic components, silica, and TOC from the raw water supply [260]. IPA is commonly used to evaluate RO membranes as a model substance for TOC removal [254]. Urea is also a troublesome organic substance in UPW production. During March and April of 2001, the Intel semiconductor manufacturing facilities that obtained feedwater from the City of Hillsboro, OR, experienced multiple TOC excursions in the final purified UPW supplied to the fab [255,256]. When TOC increases were observed, trace amounts of organic matter were analyzed, and it was found that urea, presumed to originate from agricultural production activities, was the main cause of the TOC increase. The usual UPW purification processes, including AC, RO, ion exchange, ozonation, UV, etc., were ineffective at removing urea. Weerakoon et al. [261] pointed out that due to the low removal efficiencies for RO/NF membranes, significant improvements in current membranes would be required before their application becomes feasible.

Boron removal is of critical importance in both seawater desalination and UPW production. Low boron concentrations (e.g., 0.3–0.5 mg/L or less) may be required for irrigation applications such as citrus fruits and semiconductor manufacturing. In order to meet this requirement, two-pass RO may have to be used, but to increase the boron rejection, NaOH is added to the first-pass permeate, raising the pH to about 10. Thus, there is a need to develop RO membranes for seawater desalination (SWRO) and brackish water desalination (BWRO) with a high boron rejection to reduce alkali consumption and O&M costs.

In recent years, interest in removing micropollutants by RO has increased due to the stringent drinking water quality standards and the application of wastewater reuse for drinking purposes, i.e., potable reuse. Among these micropollutants, N-nitrosamines, in particular NDMA, 1,4-dioxane, and THMs, are challenging to remove effectively by RO due to their low molecular weights. NDMA is formed as an unintentional byproduct of the chlorination of wastewater and drinking water at treatment plants, especially where chloramines are used for disinfection [262]. NDMA has been reported to exhibit lower rejection compared to other N-nitrosoalkylamines. For example, the ULP membrane (ESPA3) showed 54% rejection at 150 psi, while the LP RO membranes had 61% (BW30) and 70% (LFC3) rejection at 225 psi [263].

Regarding the action as a rejection enhancer, most LMWOC foulants are thought to function as rejection enhancers [115,137,194,264,265,266], but the key points for selection are as follows:

- The flow rate reduction must be as low as possible.
- Targeted boron and micropollutant rejections must be increased as much as possible.
- The performance of modified BWRO membranes needs to be compared with SWRO membranes as a reference.
- The ability to be immobilized in the membrane.

**Figure 6:**
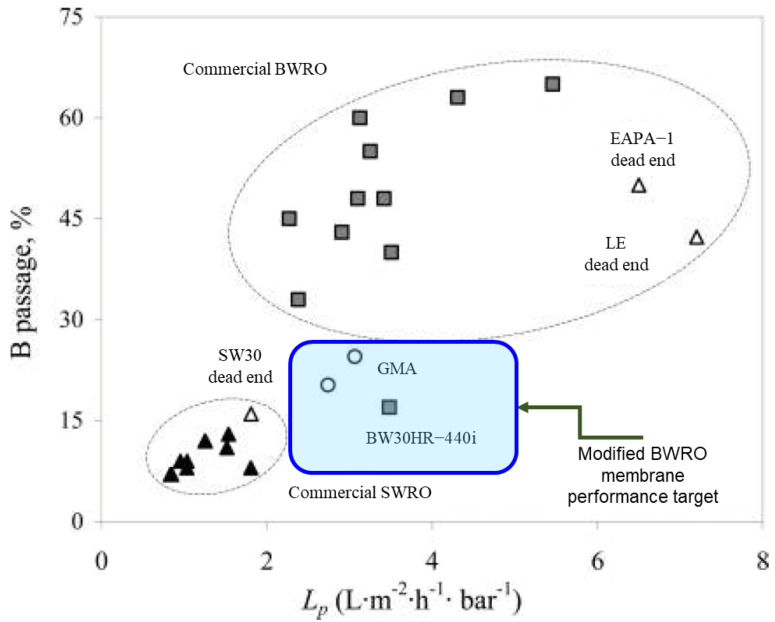
Boron passage and membrane permeability of commercial BWRO and SWRO elements. The empty circles show the results for FilmTec LE membranes modified with glycidyl methacrylate (GMA). Reprinted with copyright permission from Ref. [267] Copyright 2011 American Chemical Society (The author added the target area for the modified BWRO membrane).

Bernstein et al. [267] examined membrane modification by concentration polarization-enhanced radical graft polymerization using a diluted aqueous solution of appropriate monomer such as glycidyl methacrylate (GMA) to improve boron rejection. Figure 6 compares different commercial SWRO and BWRO membranes and GMA-modified FilmTec LE membranes. There is a large difference in boron removal performance between SWRO and BWRO membranes. SWRO membranes having small pore sizes exhibit higher boron removal performance. SWRO membranes are used as the base membrane when modifying membranes for seawater desalination. On the other hand, the modification of BWRO membranes is required for UPW production and two-pass RO. When modifying BWRO membranes with LMWOC, it is necessary to confirm that the permeability is higher than that of SWRO, as it would be preferable to simply use SWRO membranes without treatment.

For the immobilization of LMWOC, the methods mentioned in the low-fouling membrane section can be applied, with diazonium modification considered particularly useful.

#### 7.1.1. Tannic Acid (TA)

TA is the most investigated organic compound as a membrane performance enhancer and RJA. It was reported that every B-9 and B-10 “Permasep” RO permeator was posttreated with PT-A (polyvinyl methyl ether, PVME) during manufacturing. B-10 permeators were also posttreated with PT-B (TA) during manufacturing [268]. New B-10 permeators typically required treatment with PT-B after the initial flushing prior to being placed on stream. It was discovered that semipermeable membranes having permanently reduced salt passage are obtained by contacting membranes with solutions of hydrolyzable tannins [269]. However, the rapid loss of PT-B was observed at a seawater desalination plant (Ghar Lapsi seawater RO facility in Malta) [270,271]. Adding hydrolyzable tannin solution to seawater forms a precipitate, aggravating the permeator’s fouling [272]. This problem was resolved by applying the PT-B treatment at a lower pH [270,272].

TA treatment is also effective for TFC PA RO/NF; examples of such treatments are summarized in Table 7. The tannins examined were mainly hydrolyzed tannins with molecular weights ranging from 900 to 3000 [256]. As the MW of TA is relatively large, a prominent effect is seen in ULP membranes and NF membranes having larger aggregate pores.

Sato and Tamura comprehensively investigated TA treatment [240,284,287]. The effect of tannins on NF membranes was evaluated using various tannins (hydrolyzed, condensed, synthetic tannins, etc.). As a result, they found that Chinese gallnut had the highest effect on the separation performance of LES90 NF membrane, e.g., conductivity, Ca, and silica passage. The TA treatment also effectively improved RO rejection performance, including conductivity, Ca, silica, and IPA rejections. They studied the impact of treatment mode, concentration, and time on this effect.

Precipitation may occur when used at high concentrations or when polyphenols other than gallnut are used. Thus, it is necessary to lower the pH by adding an organic acid such as citric acid [274]. It was found that permeation treatment under pressure was superior to submersion treatment [275]. Treatment with more than 5 mg/L of tannic acid for 10 min was required to improve separation performance [240]. In addition, because hydrolyzed tannin is a natural product, there can be significant differences in performance after treatment depending on the lot, so care must be taken [278].

Mitrouli et al. [279] examined the same DuPont PVME and TA posttreatment methods for ULP and LP RO membranes. The treatment of a pristine ULP RO membrane with 10–30 ppm PVME followed by 10–30 ppm TA significantly improved membrane rejection performance. However, a similar treatment of LP RO, which exhibited good initial salt-rejection performance, did not produce any further improvements.

Another example of TA treatment is its use as a rejection-enhancing agent in producing NF membranes for water softening [281,282]. The membrane can be prepared by treating a PA RO membrane with a strong mineral acid such as phosphoric acid, followed by a rejection-enhancing agent (TA or a hydrolyzed tannin). Under transmembrane pressures of 50 psi, these modified membranes can achieve a magnesium sulfate (MgSO_4_) rejection exceeding 90% for 0.2% aqueous magnesium sulfate solution.

Furthermore, TA treatment can improve the chlorine and oxidant tolerance of RO/NF membranes. Moch et al. explained this action for DuPont’s aramid membrane: chlorine first reacts with TA before attacking the membrane [165,289]. Sato and Tamura [287] evaluated the effect of TA on preventing oxidative damage in commercial RO/NF membranes. They investigated the impact of periodic TA (gallnut) treatments on separation performance and flux in membranes with oxidative damage. The treated NF membrane (LES90) showed good tolerance to oxidants at stable flux and stable separation performance in the same manner mentioned by Moch [165]. However, when they extended their study to ES10 ULP RO membrane, they found that while TA treatment showed improved oxidant tolerance, the treatment was less effective than in the case of LES90.

When the raw water has an oxidizing atmosphere with a high oxidation–reduction potential (ORP) value, treatment with TA alone proved ineffective. It was speculated that TA is decomposed prior to reaching the membrane. In such cases, rejection performance can be sustained by intermittently supplying pressurized water containing polyphenols (TA) and a reducing agent such as SBS [288].

#### 7.1.2. Other LMWOCs as Performance Enhancers

It is known that when municipal wastewater effluent is treated with RO, the rejection of LMWOCs increases due to fouling. Xu et al. [290] observed that membrane fouling with secondary effluent resulted in rejection enhancement for chloroform, bromoform, and trichloroethylene. Similarly, N-nitrosamine rejection increased when the membranes were fouled by tertiary effluent. The rejection of LMW N-nitrosamines, such as DMNA, was most affected by membrane fouling [291]. The characterization of organics in the treated wastewater samples revealed that increased NDMA rejection could be caused by foulants composed of LMWOCs (<300 Da) [292]. On the other hand, membrane fouling caused by large model organic foulants (e.g., sodium alginate, bovine serum albumin, and HA) does not significantly impact NDMA rejection [293].

These cases indicate that membrane modification with LMWOC can improve the rejection of non-dissociated organic and inorganic substances. Although not an in situ treatment, the membrane performance can be enhanced by contacting various reactive organic substances after the interfacial polymerization of polyfunctional amines and acid chlorides [294].

**Figure 7:**
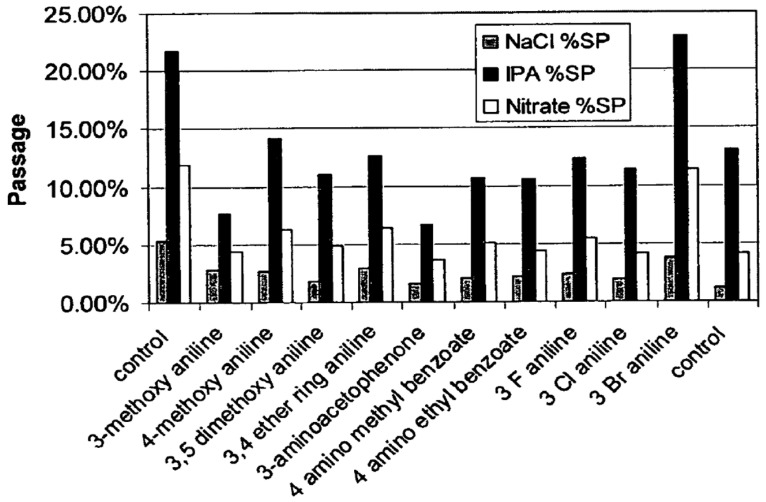
Sodium chloride, IPA, and nitrate passage in membranes modified with phenyl amine compounds [294].

Figure 7 shows an example of an improvement in IPA rejection by treatment with phenyl amine compounds showing that most amine-modified membranes reveal IPA passage of 10–12%. However, the IPA passage for the membrane modified with 3-aminoacetophenone and 3-methoxyaniline decreased to about 7%. Therefore, screening various LMWOCs may make it possible to find a suitable enhancer that balances flow rate and rejection improvement. Table 8 summarizes the examples of LMWOCs examined for enhancing RO membrane performance reported.

Improvement of the salt rejection performance of RO membranes is required in various situations as follows:

- Improvement of salt rejection by plugging micro defects;
- Improving the rejection of monovalent cations at low concentrations;
- Improvement of salt rejection at low and high pH.

TA treatment has been reported to plug micro defects and aggregate pores and improve the rejection performance. As a sealant, cationic organic substances that can be immobilized through electrostatic interactions with the membrane are considered. Rejection performance was enhanced by treating PA asymmetric membranes with cationic dyes (crystal violet and malachite green) [295].

In UPW production, low total dissolved solids (TDS) water supplied from the first-pass RO permeate (in a double-pass RO system) or ion exchange effluent may be treated with RO. In this case, there is a problem in that the rejection of monovalent cations such as Na ion decreases due to the Donnan effect [296,297]. Attempts have been made to modify the membrane surface to be positively charged to improve the rejection of Na ions [298]. A simple method is to treat it with polycations such as PEI or cationic flocculants. Similar effects were observed with membranes treated with LMW quaternary ammonium compounds such as CTAB. However, performance longevity needs to be verified. Meanwhile, treating the membrane with PEG increases TOC rejection, and the specific resistance of the permeate in the 2-pass RO was also improved [299].

**Table 8:** Summary of LMWOC performance-enhancing agents.

Targets	Rejection Enhancer	Comments	Reference
***Improvement of inorganic and organic rejections***			
Na+ ion	Quaternary ammonium	Under low TDS conditions	[298]
Salt rejection	Cationic organic compounds	Asymmetric polyamide membrane	[295]
Urea, IPA	Polyalkylene glycol	PEG (MW 2000–6000)	[300]
Salt rejection	Polyalkylene glycol	PEG (MW 1000–10,000), low pH performance	[301]
TOC	Polyalkylene glycol	Improve TOC rejection at high pH (>9.5)	[302]
Salt rejection	Polyalkylene glycol	Under low TDS conditions, i.e., second pass RO	[299]
IPA	Polyalkylene glycol + LBL PEC	After PEG treatment, an LBL PEC treatment	[303]
As(III)	Aramide dendrimers	With oligoethylene glycol chains, for NF membrane	[230]
Boron	Surfactants	Cationic, nonionic, and anionic surfactants	[266]
Boron	Pyrogallol derivatives	Pyrogallol and/or a pyrogallol derivative: MW < 500	[304]
Boron	Higher alkylamines	Decylamine and dodecylamine	[305,306,307]
Boron	4-Nitrobenzenesulfonyl chloride	Hydrolyzed to 4-Nitrobenzenesulfonic acid	[308]
Urea	MPD	Carbodiimide chemistry is used to attach a diamine	[309]
Urea	MPD, 1,8-diaminooctane, etc.	Carbodiimide chemistry and effect of amines	[310]
***Improvement of Micropollutant rejection***			
NDMA	Tryptophan	Model LMW foulant in municipal wastewater	[292]
NDMA	Alkylamines	Hexylamine, decylamine, dodecylamine, etc.	[311,312]
***Improve chlorine tolerance***			
Chlorine	Diphenylamine	First, a membrane is treated with sodium peroxide.	[313]

Commercially available PEG has a wide range of MWs from 300 to 100,000 Da. As mentioned in Part 1, PEG with an MW of more than 1000 acts as a significant foulant. Kawakatsu et al. [299,300,301,302,303] intensively investigated its use as a performance enhancer for RO membranes. In particular, PEG with an MW of several thousand has positive effects on membrane modifications, as shown in Table 8. In addition to improving the rejection performance of uncharged boron and urea, TOC at high pH and salt rejection at low pH are increased. With normal APA RO membranes, the salt rejection decreases as the pH of the feed solution approaches the isoelectric point. In this case, PEG treatment effectively improves rejection in the low pH range (pH 4–7) [301]. However, PEG treatment also seems to have issues with long-term performance stability. For example, it was shown that the IPA removal performance of sulfonated PEG-treated RO membranes gradually decreases with treatment time. To solve this problem, they discovered a method to form a PEC protective layer using the LbL method (alternate treatment with polyvinylamidine and PSS-Na) after sulfonated PEG treatment [303].

The application of various LMWOC foulants (nonionic and cationic surfactants, pyrogallol derivatives, etc.) has also been considered to improve the rejection of non-dissociated inorganic and organic substances. Among these, higher alkylamines, i.e., decylamine and dodecylamine, show a prominent effect [306,311]. Fujioka et al. [311] evaluated the effect of alkyl chain length, i.e., hexyl, octyl, decyl, and dodecyl. The NDMA rejection increased with alkyl chain length from 55% to 82% (the pristine membrane: 42%). Along with rejection improvement, the permeate flux was decreased.

An interesting treatment method was reported: the membrane is treated while swelling with an appropriate solvent instead of contacting it with LMWOC in an aqueous solution (swelling–embedding–shrinking, SES) [308]. The enhancer easily penetrates the inside of the membrane due to swelling. After that, the PA network shrinks due to the evaporation of the solvent, and the enhancer is fixed to the PA network. An example was reported in which ethanol was used as the solvent, and 4-nitrobenzenesulfonyl chloride (NBS) was used as the enhancer. After treatment, NBS is hydrolyzed into sulfonic acid in water. This method increased the boron rejection from 82.12% to 93.10%.

As an immobilization method for the rejection-enhancing agents, carbodiimides were used to activate residual carboxyl groups in the PA layer and form amide bonds with MPD. By this method, MPD or any other amines can be immobilized [309].

Next, improved performance is reviewed. Table 9 summarizes the performance changes after various posttreatments.

All the enhancers examined improved the rejection performance of uncharged LMW substances, but the following substances showed remarkable effects:

- PEG-based: PEG (MW 1000–10,000) and Tween 80 (ethoxylated surfactant);
- Cationic surfactant with long alkyl chains: CTAB and dodecylamine;
- NBS: SES method.

Both PEG and cationic surfactants are linear molecules. In order to improve the rejection of LMW substances such as boron, urea, and NDMA, it may be necessary to plug smaller pores beyond aggregate pores. For this purpose, linear molecules having small molecular widths may be more suitable. Although seawater desalination (SWRO) membranes are thought to have a small pore size that results in high boron rejection, the dodecylamine treatment reduced the flow rate by 60%, suggesting that they may penetrate smaller pores as well. In addition, the SES method may be effective for fixing the enhancing agent in smaller pores.

Next, performance changes by enhancers and the oxidation treatment with bromine or iodine in SWRO membranes are compared for boron removal. It has been observed that the oxidation with bromine or iodine causes a significant decrease in flow rate during seawater desalination. However, it is considered to be used as a performance enhancer, just like LMWOC foulants. Table 10 shows a comparison of the performance of the two. Dodecylamine and NBS reduce the flow rate by about 60%, iodine chloride reduces the flow rate by about 50%, but other bromine and iodine treatments reduce the flow rate by about 30–40%.

In some cases, boron rejection after treatment exceeds 95%. However, since bromine is a strong oxidizing agent, the RO membrane may be deteriorated. On the other hand, it was reported that iodine does not break amide bonds [317], so iodine may be more suitable when used as an enhancer. Furthermore, since it is known that prechlorination can minimize the flow losses by bromine and iodine [318,319], performance enhancement could be optimized by combining the prechlorination followed by bromination or iodization. As such, bromine and iodine are significantly effective in improving boron rejection, so when applying an LMWCO enhancer, a comparative study with bromine or iodine treatment may be necessary.

Regarding the modification of BWRO membranes, all of the BWRO data shown in Table 9 use ULP-type membranes as the base membrane, so to determine its effectiveness, it is necessary to compare it with ULP membranes treated with halogens or untreated LP and SWRO membranes. Figure 6 shows LP RO (BW30HR) has better boron rejection than the modified ULP RO (LE) membranes.

### 7.2. Application as an RJA

García et al. [320] reported summarized results from their autopsy analysis of over 500 SWRO membranes conducted over a 20-year period. Although the main issue on SWRO membranes is fouling (63%) for performance failure, oxidation (9%) and abrasion (28%) are cited as other critical causes. Performance decline with fouling can generally be restored by cleaning, but membrane deterioration due to oxidation and abrasion cannot be restored. In such cases, the need for chemicals and treatment methods called “rejuvenation” that can restore membrane performance is recognized. Rejuvenation is the enhancement of salt-rejection characteristics by applying chemical treatments to the surface of semipermeable membranes [135]. Rejuvenation techniques were studied by membrane manufacturers and the government during the 1970s and early 1980s and were applied to CA and PA hollow fiber membranes. Amjad et al. [135] summarized the rejuvenation techniques as follows:

- Two mechanisms are proposed to explain the rejuvenation process: “surface treatment”, which is, in effect, a surface coating on the membrane, and “hole plugging”.
- Membrane rejuvenation typically increases salt rejection to at least 94%.
- The key to successful application is to clean the RO membranes thoroughly.
- When salt rejection is below 75%, successful rejuvenation is unlikely.

DuPont’s most common rejuvenation technique was first applied to their B-9 and B-10 permeators, that is, PT-A and PT-B treatment [268]. B-10 permeators usually had to be treated with PT-B before placing on-stream after the initial flushing and after any cleaning operation. It was reported that the train salt rejection at an actual plant in India increased from 91.5% to 98.6% with PT-B treatment during commissioning [321]. PT-A and PT-B treatments were implemented in many plants, and the following findings have been drawn [135]:

- PT-B treatment should be conducted at a pH less than 5.0 [272].
- PT-A is generally durable.
- PT-B wears away with time.
- Cleaning treatments may remove PT-A and will readily remove PT-B.
- It is usually very effective when salt rejection is above 80%.

PT-B is relatively insoluble in seawater and forms a brown precipitate even at a PT-B concentration of only 10 mg/L [272,322]. If the seawater pH is less than 5.0, no precipitate is formed even at a PT-B concentration of 80 mg/L. It was also noted that the pH required to maintain a stable or controllable salt passage is site-dependent and is normally determined experimentally [271].

The later developed TFC RO membrane had sufficient initial performance. Although the membrane’s durability was weak against chlorine, it was sufficient if carefully managed, so less attention was paid to rejuvenation. However, in recent years, the thought of using products for as long as possible has become widespread regarding sustainable manufacturing practices. RO membranes can be used in some fields even if their rejection performance drops slightly, and in such fields, rejuvenation can extend the membrane’s lifespan and reduce operating costs. NF membranes, with lower salt rejection requirements, are suitable for rejuvenation treatment. TA has been extensively studied as a TFC RO/NF membrane RJA [240,323].

#### 7.2.1. TA and Polyphenols as RJAs

Treatment with Chinese gallnut TA (MW 1700 Da) can improve the performance of the APA NF (LES90) membrane with a salt rejection of 95% [240]. Assuming this NF membrane is a model RO membrane that is oxidatively deteriorated, it is possible to assume that TA, which has a much larger MW than the RO membrane MWCO, is effective for rejuvenation. This idea is evident from the results of a modification test of NF membranes using three types of polyphenols conducted by Pan et al. [324]. Pore size distributions estimated by PEG fractionation were unchanged for NF membrane treated with protocatechuic acid (MW 154.1 Da) from those of untreated membrane. On the other hand, the proportion of large pores with a diameter of 0.4 to 0.7 nm was significantly reduced by treatment with TA or tea polyphenols, and the pore size distribution became sharper.

Some examples of TFC RO membrane performance recovery using TA are summarized in Table 11. Case 1 is an early example of TA treatment, in which the tests were performed at the Sabha BWRO desalination plant in Israel [325]. The original RO elements were in operation for over two years, and the actual salt passage deteriorated from the initial level of 2–3% to 15–20%. The first TA treatment with 5 ppm for 10 min was conducted after acid (citric acid, pH 4) and alkaline cleaning (NaOH, pH 10.5). This treatment resulted in a decrease in salt passage by 55% and productivity by 22%. Next, the TA dosing was decreased to 1 ppm, resulting in a more moderate decrease in productivity and salt rejection. Based on those results, a cleaning and rejuvenation treatment was applied to twelve old spiral wound elements with 1 ppm of TA for 15 min. An average reduction of 43% of the salt passage was obtained, as shown in Table 11. This plant also used polyamide hollow fiber membranes, which required 100 ppm TA treatment. In comparison, it is noteworthy that TFC PA membrane rejuvenation was effective with low concentrations of TA treatment.

Case 1 is an example of a substantial effect. However, there are also cases where the effect was not as significant, depending on the degree of deterioration and membrane type [240]. When RO elements were oxidized by chlorine, salt passage increased from 1.5 to close to 10%. A trial with a commercially available TA derivative initially showed promising results: the salt passage decreased from 10% to 7%. However, this effect was lost a couple of hours after dosing. The first assumption was that the membranes were not clean enough or there was still residual chlorine in the feed. Therefore, a caustic CIP was performed. The result of the second coating trial with the tannin product was not much better [327].

As observed in DuPont’s PT-B treatment, the rejuvenation efficacy for TFC PA membranes is also affected by the treatment conditions (effect of pre-cleaning, pH, concentration, treatment pressure, etc.). First, regarding the effect of pH, it is important to treat at a low pH to increase the solubility of TA, but Silva et al. [323] reported data showing that the effect decreases at pH 2–2.4. Good results are obtained at pH 3–5. On the other hand, Sato and Tamura [283] reported that enough modification effects were observed even at pH 2.2.

Case 2 in Table 11 examined the effect of pressurization [283]. Although sufficient treatment results were obtained under pressurized conditions at a permeate flux of 41.7 LMH, performance did not recover after one hour of immersion treatment. Meanwhile, no difference in treatment effect due to pressure was observed when the treatment pressure was in the range of 0.196 to 0.785 MPa [323].

Case 3 shows data on the importance of pre-cleaning [326]. A deteriorated membrane was cleaned with a 0.2 wt% citric acid solution and a pH 11 sodium hydroxide solution for 6 h each, and then TA treatment was performed. When cleaning was not performed, performance recovery was not satisfactory. All the examples in Table 11 are cleaned with alkali just before TA treatment. Since the membrane, which was deteriorated by chlorine and other oxidative agents, swells when cleaned with alkali, the TA molecules are incorporated into the polymer matrix of the membrane, which may result in a better effect, as with the SES procedure. Concerning the effect of concentration, a sufficient effect was obtained even at 1 ppm (case 1), but in other cases, the concentration is quite high at 100–240 ppm. No difference in effect was observed in the range of 80 to 400 ppm [323].

To evaluate how long the TA rejuvenation treatment can last, the chlorine-degraded BW30 spiral wound module was treated as shown in case 4 of Table 11 and evaluated for 30 days [323]. The salt rejection remained almost constant during the 30 days of evaluation, with 97.6% at the beginning and 97.0% at the end. The silica rejection decreased during the first 4 days of evaluation, from 96% to 91%, and then remained relatively constant for the subsequent 26 days. However, the loss of tannic acid from the membrane surface was also evidenced by an increase in permeate flux. A method has been disclosed to alleviate the problem where the TA-treated membrane is contacted with an aqueous solution containing heavy metals (potassium antimonyl tartrate, Iron(III) hydroxide, etc.) [277]. The membrane posttreated with heavy metals maintained its initial performance even after one week, whereas rejection performance deteriorated without posttreatment. It is reported that polyfunctional tannins with several o-dihydroxyphenyl functional groups are good chelators that can form precipitates with metal ions [328]. Therefore, it is presumed that the TA treatment with heavy metals after TA treatment insolubilizes the TA and stabilizes its performance.

Posttreatment with polycation solution can be another method to immobilize TA. TA can be combined with a polycation, such as PEI, chitosan, etc., to form a PEC. Chitosan–TA complex treatment was reported to increase salt rejection up to 33.17% on oxidatively degraded RO membrane [329].

#### 7.2.2. Other RJAs

To avoid excessive permeate flow rate reduction, bulky hydrophobic or amino organic substances with relatively large MW (e.g., more than 300 Da) are preferable for rejuvenating oxidatively or chemically damaged membranes, but there are few reports of such use.

Water-soluble amines were reported to effectively rejuvenate RO membranes consisting of crosslinked polymers based on furfuryl alcohol [330,331]. Water-soluble amino compounds include LMW alkyl, aromatic, and alicyclic amine compounds (e.g., sulfamic acid, sulfanilic acid, and diaminobenzoic acid) and high MW compounds such as PEI. A remarkable salt rejection improvement was observed with diamino-diphenylmethane (MW 198.3 Da, log *p* = 1.59) among LMWOCs.

Examples of nonionic surfactants and PEG as RJAs, other than TA, examined in TFC RO are shown in Table 12. Triton X-405 (MW 1974.47 Da, HLB = 17.9) is an octylphenol ethoxylate having long ethoxy chains (n = 40). Compared to cetyltrimethylammonium chloride (CTAC), Triton X-405 does not cause a significant decrease in flow rate and can improve silica rejection from 98% to 99% [332].

As mentioned in the subsection on performance enhancers, PEG is also useful as an RJA. Its effectiveness depends on several factors, like TA treatment (MW, concentration, pressure conditions, pre-cleaning, and pH) [333]. First, regarding the effect of MW, an MW of about 1000 Da does not restore boron rejection performance, and an MW of about 6000 to 100,000 Da is considered suitable. Immersion treatment is ineffective. In addition, performance recovery is improved by treating deteriorated membranes after alkaline cleaning [334]. However, unlike TA, PEG can be used at high pH, and the effect is also significant in that case, so it can be assumed that the swelling of the membrane is effective in the penetration of PEG molecules. In regular membrane cleaning, the rejection and flow rate can be returned to normal by adding an acid cleaning after alkaline cleaning, so it may be more effective to add an acid cleaning based on the SES concept.

#### 7.2.3. Multicomponent RJAs

**Figure 8:**
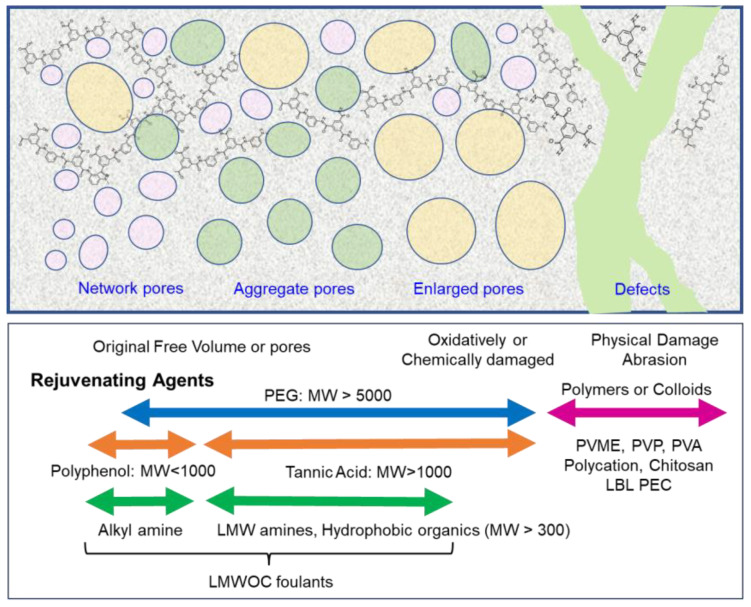
Schematic image of a degraded membrane and proposed RJAs.

As shown in Figure 8, RO/NF membranes are physically damaged by abrasion caused by foreign matter, compaction during operation, etc., resulting in membrane defects. In addition, oxidative attacks cause polyamide chains to break, forming larger pores and pinholes, which deteriorates rejection performance. When rejuvenating such deteriorated membranes, a single component, such as PEG and TA, may not be sufficient. For example, in rejuvenating SWRO membranes, the recovery of boron rejection is essential, but TA treatment alone is insufficient to plug smaller-sized pores; thus, the improvement of boron rejection is limited. Nonionic surfactants can plug aggregate pores but cannot repair large pores or pinholes. Therefore, one of the measures to solve this problem is to use a multicomponent RJA instead of a single component.

As a two-component RJA, DuPont proposed a two-step treatment using PT-A (PVME) and PT-B (TA). PVME covers defects on the membrane surface, and TA treatment can plug pores. This method was developed for rejuvenating polyamide hollow fiber membranes, but Valls et al. [335] developed a technique suitable for TFC PA membranes. However, a problem encountered with the PVME solution is that it cannot be used for online treatment with warm feed waters due to its low critical solution temperature (cloud point) of about 34 °C to 37 °C. Instead of PVME, Mitrouli et al. [336,337] investigated the effectiveness of PA RO membrane treatment by PVP and PVA, followed by TA. They applied these polymers to degraded membranes by dead-end and cross-flow modes. There was significant rejection improvement, with a modest concurrent flux reduction. The durability of coatings with all three RJAs was further evaluated by extending the operation of the cross-flow unit for approximately one month. The improved membrane performance remained unchanged.

This stable membrane performance may be due to the formation of a complex between the polymer and TA by contacting TA after PVP or PVA treatment, which insolubilizes the water-soluble polymer. Various tannin-complexing agents, such as PVP, PEG, and chitosan, have been reported. PVP and PEG form a hydrogen-bonded complex with TA [338]. Raval et al. [329] demonstrated an increase in salt rejection of up to 33.17% of oxidatively degraded RO membranes by chitosan–TA complex treatment. Meanwhile, PEG is also known as an effective RJA. Thus, TA treatment following PEG treatment may improve performance stability through complex formation.

Next, Kawakatsu et al. [339,340] reported a three-component RJA. Their strategy to rejuvenate the deteriorated membrane is to attach small molecules with amino groups to the carboxyl groups at the damaged portion and cover them with larger molecules.

The three-step covering method was developed as a rejuvenation technology using different MW chemicals. The No. 1 agent has a smaller MW than the No. 2 and No. 3 agents. The No. 1 agent can plug small holes in the membrane. Then, the No. 2 agent is a middle MW, so it deposits onto the No. 1 agent and covers the large hole. Lastly, the No. 3 agent can enhance the stability of the coating. In terms of these RJAs, they proposed several combinations. Table 13 shows some examples of these three-component compounds. The first example is a three-component system consisting of aminopentane, 3,5-diaminobenzoic acid, and polyvinylamidine, all of which have amino groups [341]. Compared to single- or two-component treatment, the three-component treatment is superior in improving salt rejection. In addition, no difference was observed in the permeation flow rate after treatment. The next example is a three-component system of arginine–aspartame–TA [342]. In either single-component case, the IPA rejection was insufficient, but a remarkable improvement in IPA rejection performance of over 90% was observed with the three-component system. This component is also safer for the environment and humans and makes it easy to dispose of waste liquid, so it is suitable for on-site treatment.

Figure 8 summarizes the potential RJA for deteriorated TFC RO/NF membranes. PEG is a linear molecule that is effective in improving boron rejection performance, so two components, PEG treatment followed by TA treatment, may be sufficient. TA plays two essential roles in rejuvenation, i.e., plugging large pores and immobilizing water-soluble polymer coating. Thus, after treating the deteriorated membrane with a mixture of LMWOC (amines, nonionic surfactants, phenolics, etc.) and water-soluble polymer (PVP, chitosan, etc.), TA treatment may make it possible to stabilize the RJA and enhance rejection performance. Future research on optimizing the RJAs and methods is anticipated.

## 8. Conclusions

Organic fouling and biofouling are significant challenges in the RO/NF treatment of low-quality water sources, including municipal and industrial wastewater. Of these, organic fouling has been considered difficult to manage. The preceding review (Part 1) [8] elucidated fouling behavior by LMWOCs and its underlying mechanisms. LMWOC fouling is an acute problem compared with other fouling phenomena.

Concerning fouling mechanisms, internal fouling typically exerts a more significant impact than external fouling. LMWOCs are trapped in larger or aggregate pores. Trapped organics impede solute permeation more than water, resulting in improved salt rejection. Two factors are critical for the entrapment of organic compounds within membrane pores: molecule size (or MW) and membrane–organic interactions, i.e., electrostatic and hydrophobic interaction. Hydrophobic interaction can be assessed using the n-octanol/water partition coefficients. Thus, molecules with higher positive log *p* values exhibit a greater influence on the decline in water flux.

The most critical LMWOC foulants commonly encountered in RO/NF plants are surfactants (especially nonionic and cationic surfactants), plasticizers (phthalates), phenolics (TCP, leachables from epoxy resins, e.g., bisphenol A, etc.), hydrocarbons, etc. Since EfOM contains such LMWOCs, initial flow loss has been a critical issue in wastewater treatment.

Sun Tzu, a Chinese military strategist, says, “If you know the enemy and know yourself, you need not fear the result of a hundred battles”. Now that we have sufficient knowledge about LMWOC fouling, it should be possible to establish effective strategies to combat it. Part 2 outlines countermeasures to solve this problem and applications using the LMWOCs.

The crucial first step in addressing the diverse challenges associated with LMWOC fouling is to accurately predict the extent of fouling. If the organic foulant has not been identified, bench testing is required. Bench testing can be performed using flat-sheet membrane testing or a small 1.8-inch diameter module used in home water purifiers. Long-term testing is not necessary to determine the extent of LMWOC fouling; typically, 3 to 4 days is sufficient.

Next, the changes in feed organics concentration and their types during operation must be monitored. However, no indicator such as SDI, which is used to predict particulate and silt fouling, can be applied to LMWCO fouling. It has been suggested that hydrophobic PA or PTFE membranes be used instead of the standard filter (mixed cellulose nitrate and cellulose acetate), which is used to measure SDI.

Once the extent of LMWOC fouling and the problematic organic compound are determined, the next step is to select a suitable pretreatment method. These include adsorbents, filters, oxidative decomposition, and antifoulants. AC, commonly used as an RO adsorbent, is also effective in removing LMWOCs. The organic removal capability varies depending on the type of AC, and AC derived from bituminous coal and peat is reported to be more effective. Direct organic removal can be achieved through oxidation treatment. Ozone treatment is effective at eliminating LMWOCs, and hybrid treatment, in which AC is installed after high pH ozone treatment, can remove foulants to a high degree. However, the clear selection criteria for these organic matter removal methods have not been established, and this may be a topic for future study.

Next, if no special pretreatment process for LMWO removal is installed, the appropriate operating method must address the problem. With LMWOC fouling, a sudden drop in flow rate occurs immediately after the start of operation. However, performance stabilizes in a short period of time thereafter, and no further drop in flow rate occurs. If the increase in operating pressure based on this initial drop in flow rate is acceptable, initial operation can be continued without taking any special measures, e.g., AC installation. As an alternate method, operation at a high pH has been proposed. By increasing the feed pH, the extent of fouling can be reduced due to increasing the membrane charge, reducing the adhesion of organic matter, and increasing the solubility of organic matter such as hydrocarbons. In addition, an antifoulant that can prevent organic fouling was also reported, but further research is required to enhance its performance.

The next step is membrane cleaning and troubleshooting. As mentioned, flow loss is sometimes irreversible or difficult to restore through regular cleaning. Thus, special CIP chemicals are necessary. These include organic solvent-based and oxidative cleaning agents. A 10% methyl cellosolve solution was used on a plant to clean the fouled membrane with a hydraulic fluid. A mixture of alcohol (30%) and EG (70%) also showed better cleaning effects at the laboratory level. An example of oxidative cleaning is the application of dichloroisocyanurate, but it could not be used continuously due to its negative effect on salt rejection. Instead, oxidative cleaning with sodium hypochlorite at high pH is a possible choice because NaOCl has a low oxidizing power at high pH. Also, if fouling due to LMWOCs is accepted as irreversible, there is no need to restore the initial performance by membrane cleaning specifically designed for LMWOC removal. Standard acid and alkali cleaning procedures can be employed.

If a significant drop in flow rate occurs immediately after the start of operation, the cause can be identified as described in the detection method. For example, by installing an AC cartridge filter, a cause of fouling can be identified by comparing the degree of fouling with and without an AC filter. In addition, membrane manufacturers and service companies frequently autopsy the problematic membrane and analyze the foulants, but LMWCO cannot often be identified by regular FT-IR analysis. In this case, the membrane is subjected to solvent extraction and then analyzed by GC-MS. Solvent extraction and solid-phase extraction methods are being implemented. In addition, pyrolysis GC-MS, which does not require extraction, seems to be a promising technique.

Until now, we have only focused on the adverse effects of LMWOC foulants, but these organic substances have the function of sealing large pores, improving membrane rejection, and restoring the performance of deteriorated membranes (rejuvenation). This review also tries to summarize these applications. Phenolics, nonionic surfactants, higher alkylamines (C10-12), etc., effectively improve the rejections of boron, urea, NDMA, etc. Interestingly, PEG with a molecular weight of 4000 or more also showed a similar effect of improving the rejection of low MW undissociated compounds. If the deteriorated membrane can be easily repaired when oxidized or physically damaged, membrane life can be extended, contributing to improved sustainability of the membrane process. Since the size of the membrane defects is not uniform, restoring performance with a single RJA is difficult. Therefore, multicomponent RJAs have been proposed.

Lastly, this review aims to provide necessary information on LMWOC fouling and its remediation. The author hopes this review can be used as a one-stop reference database when readers want to know about their LMWOC fouling problems, symptoms, mechanisms, corrective measures, i.e., prediction, pretreatment, and CIP, and ideas about low fouling membrane development.

## Figures and Tables

**Table 1:** Corrective and preventive measures for LMWOC fouling in RO/NF.

Corrective and Preventive Measures		Examples
Pretreatment	Adsorbents	Activated carbon (AC) and synthetic adsorbents
	Filters	Organic/oil removal filters
	Oxidation	Ozone, Advanced oxidation process (AOP), NaOCl, etc.
	Antifoulants	Polyvinylpyrrolidone (PVP), anionic polyvinyl alcohol (PVA), etc.
Detection	Prediction	Bench or pilot tests and coupon tests with polyamide (PA) membrane
	Identification	GC-MS (extraction or pyrolysis)
CIP	Organic CIP	Lower alcohols, butyl cellosolve, ethylene glycol (EG)/alcohol, etc.
	Oxidative CIP	Nitric acid, high pH NaOCl, dichloroisocyanurate, etc.
Operation	High pH	Negative membrane charge and swelling
	Low pH	Positive membrane charge
Low fouling membranes	Tight membranes	Seawater RO membranes
	Posttreatment	Heat treatment and nitrous acid (diazonium coupling)
	Surface modification	Polyvinyl alcohol (PVA), polyelectrolyte complex (PEC), etc.

NaOCl: sodium hypochlorite, GC-MS: Gas Chromatography/Mass Spectrometry.

**Table 3:** Oxidation and AOPs for removing organics as RO/NF pretreatment.

Oxidant	Organics	Membrane	Comments	Ref.
NaOCl	MBR EfOM	APA RO	NaOCl: 10 mg/L; Retention time: 30 min.	[57]
KMnO_4_	Surface water and EfOM	AG (RO) and HL (NF)	KMnO_4_: 0.45–0.8 mg/L	[58]
X-ray	Produced water	SWC-5	Cold-cathode X-ray: 20–30 kV/0.1–03 mA	[59]
H_2_O_2_/UV	Nonionic surfactant	Polybenzimidazole	H_2_O_2_: 100 mg/L; UV: 0.07 W·min/cm^2^	[60]
H_2_O_2_/UV	Groundwater	NF70	H_2_O_2_: 1–2 mM; UV (254 nm); TOC: 3.5 mg/L	[61]
Ozone (O_3_)	Phenolics	NF40 and FT30	Chlorophenols, O_2_ containing 2% O_3_	[62]
Ozone (O_3_)	Surface water	APA NF	O_3_: 3 ppm; Feed COD: 2–4 ppm	[63]
Ozone (O_3_)	MBR EfOM	ESPA-2	Pilot test (MBR—O_3_—RO); O_3_: 1.5–2 ppm	[64]
Ozone (O_3_)	EfOM	NF90	O_3_: 0.2 and 0.4 mg O_3_/mg DOC	[65]
Ozone (O_3_)	MBR EfOM	ESPA-DHR	O_3_: 0.8 mg O_3_/mg DOC	[66]
Ozone (O_3_)	MBR EfOM	NF90	Gas ozone concentration: 5 g O_3_/Nm^3^	[67]
O_3_ or H_2_O_2_/O_3_	NA	NTR-759 (RO)	Ozonation at high pH	[68]
O_3_ or H_2_O_2_/O_3_	NA	ES-10 (RO)	O_3_ dosing regulation (UV absorbance)	[69]
O_3_ or H_2_O_2_/O_3_	NOM, MBR EfOM	ESPA-2	O_3_: 1.5, 3, and 6 mg/L	[70,71,72]
O_3_ or H_2_O_2_/O_3_	MBR EfOM	NE90	Ozonation at high pH; O_3_: 3, 6, and 9 mg/L	[73]

**Table 4:** Summary of reported fouling and non-fouling compounds.

Organic Compound	Molecular Structure	CAS No.	Fouling	MW	Log P	Reference
Phenolic Derivatives						
2,4-Dinitrophenol	C_6_H_3_(OH)(NO_2_)_2_	51-28-5	S	184.11	1.67	[90]
2,4,6-Trichlorophenol	C_6_H_2_Cl_3_OH	88-06-2	S	197.45	3.69	[90,91]
2,4-Dicholrophenol	C_6_H_4_Cl_2_O	120-83-2	S	163.0	3.06	[90]
Phenol	C_6_H_5_OH	108-95-2	W	94.1	1.46	[90,91,92]
2-Fluorophenol	C_6_H_4_FOH	367-12-4	W	112.1	1.76	[90]
2-Nitrophenol	C_6_H_5_NO_3_	88-75-5	M	139.1	1.79	[90]
3-Nitrophenol	C_6_H_5_NO_3_	554-84-7	S	139.1	2.00	[92]
2-Chlorophenol	C_6_H_4_ClOH	95-57-8	M	128.5	2.15	[90]
Catechol	C_6_H_4_(OH)_2_	120-80-9	W	110.1	0.88	[92]
Resorcinol	C_6_H_4_(OH)_2_	108-46-3	W	110.1	0.8	[93]
Pyrogallol	C_6_H_3_(OH)_3_	87-66-1	M	126.1	0.23	[92]
Phloroglucinol	C_6_H_3_(OH)_3_	108-73-6	W	126.1	0.16	[93]
3-Hydroxybenzonic acid	HOC_6_H_4_CO_2_H	99-96-7	M	138.1	1.58	[93]
Bisphenol A	(CH_3_)_2_C(C_6_H_4_OH)_2_	80-05-7	S	228.3	3.64	[94]
Nonionic Surfactant						
Triton-X 35	C_14_H_22_O(C_2_H_4_O)_3_	9036-19-5	S	340	4.31	
Triton-X 45	C_14_H_22_O(C_2_H_4_O)_4_	2315-63-1	S	382.5	4.04	
Plasticizer						
Tributyl Phosphate	(C_4_H_9_)_3_PO_4_	126-73-8	S	266.3	4.0	[95]
Dimethyl terephthalate	C_6_H_4_(COOCH_3_)_2_	120-61-6	S	194.2	2.25	[23]
Dibutyl Phthalate	C_6_H_4_(COOC_4_H_9_)_2_	84-74-2	S	278.3	4.61	[23,96]
Other Aromatics						
Alkylated diphenylamine	C_20_H_27_N	68921-45-9	S	281.43	5.2	[97]
Diphenylamine	(C_6_H_5_)_2_NH	122-39-4	S	169.22	3.29	[97]
Cumene hydroperoxide	C_6_H_5_C(CH_3_)_2_OOH	80-15-9	S	152.19	2.41	[98]
Benzene	C_6_H_6_	71-43-2	W	78.11	1.99	[90]
Aliphatic Organics						
Methanol	CH_3_OH	67-56-1	W	32	−0.63	
Ethanol	C_2_H_5_OH	64-17-5	W	46	−0.14	
2-Propanol	CH_3_CH(OH)CH_3_	67-63-0	W	60	0.05	
Ethylene glycol	C_2_H_4_(OH)_2_	107-21-1	W	62.07	−1.36	
Propylene glycol	CH_3_CHOHCH_2_OH	57-55-6	W	76.1	−0.92	
Acetic acid	CH_3_COOH	64-19-7	W	60	−0.17	[91]
n-Octanoic acid	CH_3_(CH_2_)_6_COOH	124-07-2	M	144.21	3.05	[99]
n-Hexane	C_6_H_14_	110-54-3	W	86.18	3.9	[100]
Glucose	C_6_H_12_O_6_	50-99-7	W	180.2	−3.24	
Sucrose	C_12_H_22_O_11_	57-50-1	W	342.3	−3.7	

S: strong fouling compound; M: moderate fouling compound; W: weak fouling compound.

**Table 5:** Cleaning agents and methods reported to be effective for LMWOC fouling.

CIP Solution	Components and Comments	Reference
***Alkaline-based Cleaner***		
Pressurized cleaning	A part of the alkaline solution is passed through fouled membranes.	[138]
Alkaline cleaner	An aliphatic or aromatic amide-containing cleaner.	[139]
Alkaline cleaner	Periodical change from an amide cleaner to no amide-containing one.	[140,141]
***Organic Cleaner***		
Isopropyl alcohol	IPA	[135]
Methanol	30% MeOH	[23]
Butyl cellosolve	10% butyl cellosolve (ethylene glycol monobutyl ether)	[142,143]
Polyol/alcohol	A mixture of Polyols (such as EG and PG) and alcohols (e.g., MeOH)	[103,144,145,146]
Organic Solutions	50% MeOH or 10% ethylene glycol monobutyl ether	[147]
***Oxidative Cleaner***		
Nitric acid	High-concentration nitric acid	[118,137,148]
Nitric acid	Three step cleaning: alkaline (pH > 12) + nitric acid (pH < 1) + alkaline (pH > 12)	[149]
Polyol + nitric acid	Two-step cleaning: Nitric acid cleaning followed by EG cleaning	[150]
High pH NaOCl	Cleaning solution containing oxidizing substances with higher pH.	[151,152,153,154,155]
High pH NaOCl	NaOCl concertation < 10 mg/L as free chlorine and pH > 10	[156]
H_2_O_2_ + NaOCl	An inorganic peroxide cleaning and rinsing with an alkali metal or alkaline earth metal hypohalite.	[157]
Oxidizing agent	An oxidizing agent, followed by cleaning with an alkaline detergent	[136]
Dichloroisocyanurate	Flow was restored, but there was a negative effect on salt rejection.	[137]
H_2_O_2_	Alkaline H_2_O_2_ (pH12)	[155,158]
Chloramines	Chloramine compounds, e.g., monochlorosulfamic acid at high pH	[159]

**Table 6:** LMW compounds for requiring improved rejections.

Components	MW	Applications
***Neutral organic and inorganic compounds***		
IPA	60.1	UPW [254]
Urea	60.06	UPW [255,256]
Boron: B(OH)_3_	61.83	UPW and seawater desalination [257]
Arsenite, As(III): H_3_AsO_3_	125.9	Drinking water treatment
***Micropollutants***		
NDMA	74.08	Municipal wastewater reuse
1,4-dioxane	88.11	Municipal wastewater reuse
Trihalomethanes (THMs)	119.37 (CHCl_3_)	Drinking water treatment (DBP removal)
Haloalkane, haloalkene, etc.		Drinking water (volatile organic compounds) [258,259]

**Table 7:** Summary of TA treatment for TFC RO/NF membranes.

Purpose	Polyphenols	Treatment Method and Comments	Reference
***RO***			
Rej. enhancer	Tannic acid (TA)	Hot water treatment with TA;	[273]
Ca, silica, IPA	Polyphenol (gallnut)	Treatment in low pH (1–5), flushing with water;	[274,275]
Rej. enhancer	Polyphenol (gallnut)	Pressurized treatment and improving rejections at LP;	[276]
Rej. enhancer	Polyphenol (gallnut)	Optimize treatment conditions (temperature, duration);	[277]
Rej. enhancer	Polyphenol (gallnut)	Effect of polyphenol (gallnut) lot;	[278]
Rej. enhancer	Hydrolyzed tannin	Combination of PVME and TA;	[279]
***NF***			
Rej. enhancer	TA	Treatment with a strong mineral acid followed by TA;	[280,281,282]
NaCl, Ca, silica	Tannins	Treatment with various tannins	[240,283,284]
Other applications (improve oxidant tolerance)			
Anti-oxidant	Polyphenol (gallnut)	Intermittent on-line TA (5–20 mg/L) treatment	[285,286,287]
Anti-oxidant	Polyphenol (gallnut)	TA treatment with a reducing agent (sodium bisulfite, SBS)	[288]

**Table 9:** Rejection improvements by LMWOC performance-enhancing agents.

Solutes	Rejection Enhancer	Flow (L/m^2^/h)	Rejection (%)	Passage Decrease (%)	Pressure (MPa)	Membrane	Ref.
IPA	Blank	Relative 1.0	95	–	–	ES10	[275]
	TA (gallnut)	Relative 0.95	96.5	30	–		
	Blank	44.5	77.8	–	0.75	ES20	[314]
	PEG 4000	26.0	87.9	46			
	Blank	34.2	92.3	–	0.75	ES20	[303]
	Sulfonated PEG4000 + LBL	25.4	96.7	57			
Boron	Blank (12 h run)	Relative 0.81	55.7	–	1.2	LE	[266]
	Sodium dodecylbenzene sulfonate	Relative 0.42	68.2	28			
	Tween-80	Relative 0.56	73.7	40.6			
	CTAB	Relative 0.6	70.5	33.4			
	Blank	41.7	45.1	–	–	ES20	[304]
	pyrogallol	41.7	53.7	15.7			
	Blank	Relative 1.0	84	–	5.5	TM820V	
	Propyl gallate	Relative 0.955	88	25			
	Blank	Pure water 110	74	–	5.5	SWC4B	[306]
	Dodecylamine (C12)	Pure water 45	87	50			
	Blank	34	88	–	5.5	SW30	[308]
	NBS	14	93.1	58			
Urea	Blank	42.7	15.4	–	0.75	ES20	[314]
	PEG 4000	29.1	32.2	19.9			
	PEG 7100	17.5	39.2	28.1			
	Blank (22 °C)	142	18	–	1.59	XLE	[309]
	MPD (Carbodiimide chemistry)	76	37	23			
	Blank (78 °C heat treat)	60	47	35			
	MPD (78 °C heat treat)	30	55	45			
NDMA	Blank	3.5 LMH/bar	42	–	0.42	ESPAB	[311]
	Dodecylamine (MW: 185.4))	1.2 LMH/bar	82	69	1.25		
	Dodecanamide (MW: 199.3)	2.3 LMH/bar	64	38	0.65		
	1,2-Epoxydodecane (MW: 184.3)	3.5 LMH/bar	60	31	0.42		

**Table 10:** Boron rejection comparison between LMWOC enhancer and halogen treatment.

Rejection Enhancer	Flow (L/m^2^/h)	Boron Rej. (%)	Passage Decrease (%)	Pressure (MPa)	Membrane	Ref.
***LMWOC Rejection Enhancer***						
Blank	Relative 1.0	84	–	5.5	TM820V	[304]
Propyl gallate	Relative 0.955	88	25			
Blank	Pure water 110	74	–	5.5	SWC4B	[306]
Dodecylamine (C12)	Pure water 45	87	50			
Blank	34	88	–	5.5	SW30	[308]
NBS	14	93.1	58			
***Bromine and Iodine Treatment***						
Blank	25	84	–	5.5	–	[315]
Bromine	25	92	50	6.6		
Blank	–	78	–	6.0	SWC5	[241]
Stabilized Bromine	–	96	82			
Blank	30.6	86	–	5.5	–	[316]
Br (NaOCl 100 ppm + 10 ppm NaBr)	20.1	93.6	54			
I (NaOCl 100 ppm + 20 ppm KI)	25.9	95	64			
Iodine chloride (ICl), 3 ppm	16.5	97	79			
HOI	Relative 1.0	91.96	–	5.5	SW30HR	[317]
HOI (0.013 mM·h contact)	0.7	95.33	42			
HOI (0.032 mM·h contact)	0.6	98.11	76			

**Table 11:** TA and polyphenols as RJAs.

Case	Type	Conc. (mg/L)	pH	P (MPa)	Pre- Clean	Memb.	Type	Before		After		Ref.
								Flow (LMH)	Rej. (%)	Flow (LMH)	Rej. (%)	
1	TA	1	–	2.76	AC&AL	TFC PA	Old	109% *	84	98.8% *	90.9	[325]
2	Gallnut	100	–	Yes	No	ES10	New	–	80	–	98	[283]
2	Gallnut	100	–	No	No	ES10	New	–	80	–	80	
3	Gallnut	100	2.2	Yes	AC&AL	ES10	Old	–	92	–	99.1	[326]
3	Gallnut	100	2.2	Yes	No	ES10	Old	–	92	–	93	
4	Digallic	240	4.0	0.29	AL	BW30	New	65.5	85.6	37.4	97.3	[323]

*: normalized flow, P: pressure, Pre-Clean: pre-cleaning before TA treatment, AC: acid, AL: alkaline.

**Table 12:** Other RJAs and the effect on rejection performance improvement.

Compounds	Conc. mg/L	I or Pres. (MPa)	Before			After			Ref.
			Flow (LMH)	NaCl Rej. (%)	Silica Rej. (%)	Flow (LMH)	NaCl Rej. (%)	Silica Rej. (%)	
TRITON X-405	1000	I; 16 h	76.7	99.5	98	40.8	99.6	99	[332]
CTAC	1000	I; 16 h	76.7	99.5	98	19.6	99.1	99.2	
			**Flow** **(LMH)**	**NaCl** **Rej. (%)**	**Boron** **Rej. (%)**	**Flow** **(LMH)**	**NaCl** **Rej. (%)**	**Boron** **Rej. (%)**	
PEG(1000)	15	0.45	46.3	99.5	89.1	44.6	99.6	89.5	[333]
PEG(8000)	15	0.45	46.3	99.5	89.1	37.1	99.7	92.3	
PEG(50,000)	15	0.45	46.3	99.5	89.1	36.3	99.7	92.5	
PEG(8000)	1	0.45	46.3	99.5	89.1	42.1	99.6	91.0	
PEG(8000)	15	I; 1 h	46.3	99.5	89.1	42.1	99.6	90.1	
PEG(8000)—Before CIP	–	–	28 m^3^/d	99.0	85	–	–	–	[334]
PEG(8000)—After CIP	15	0.45	32 m^3^/d	98.6	84	28 m^3^/d^3^	99.3	91	
PEG(8000)—pH 12	15	0.45	28 m^3^/d	99.0	85	22 m^3^/d	99.2	90	
PEG(8000)—No CIP	15	0.45	28 m^3^/d	99.0	85	25 m^3^/d	99.2	87	

I: immerse, CTAC: cetyltrimethylammonium chloride, CIP: pH 12 with NaOH.

**Table 13:** Three-component RJAs and the effect on rejection performance improvement.

RJAs			Before			After			Ref.
No. 1	No. 2	No. 3	Flow (LMH)	NaCl Rej. (%)	IPA Rej. (%)	Flow (LMH)	NaCl Rej. (%)	IPA Rej. (%)	
3,5-DABA	–	–	49.6	89.2	–	33.8	94.5	–	[341]
3,5-DABA	APT	–	50.0	88.4	–	34.6	95.1	–	
3,5-DABA	APT	PVAM	49.6	88.1	–	34.2	96.1	–	
Arginine	–	–	37.9	91.7	78.1	37.1	94.3	84.6	[342]
Arginine	Aspartame	–	37.9	91.2	78.3	36.7	94.7	85.0	
Arginine	Aspartame	TA	37.5	92.3	78.5	33.3	98.6	90.2	
Arginine	Aspartame	Mimosa	38.3	90.8	77.6	33.8	97.7	91.2	
–	–	TA	37.5	92.0	78.7	35.8	94.6	83.1	

3,5-DABA: 3,5-Diaminobenzoic Acid (MW 152.15 Da), APT: aminopentane (MW 87.16 Da), PVAM: polyvinylamidine arginine (MW 174.2 Da) aspartame (MW 294.3 Da).

## Data Availability

The author confirms that the data supporting the findings of this study are available within the articles, conference proceedings, patents, etc., listed in References.

## Abbreviations

2B3T	Two-bed, three-tower pure water system
2CP	2-Chlorophenol
2NP	2-Nitrophenol
3,5-DABA	3,5-Diaminobenzoic acid
4CP	4-Chlorophenol
4NP	4-Nitrophenol
AC	Activated carbon
AE	Alkyl ethoxylate
AEO	Alcohol ethoxylate
AOM	Algal organic matter
AOP	Advanced oxidation process
APA	Aromatic polyamide
APEO	Alkylphenol ethoxylate
APT	Aminopentane
ATD	Anti-telescoping device
ATP	Adenosine triphosphate
ATR	Attenuated total reflection
BAC	Benzalkonium chloride
BPA	Bisphenol A
BSA	Bovine serum albumin
BTEX	Benzene, Toluene, Ethylbenzene, and Xylenes
BWRO	Brackish water reverse osmosis
CA	Cellulose acetate
CAPB	Cocamidopropyl betaine
CAR	Carboxen
CEB	Chemical-enhanced backwash
CF	Cartridge filter
CIP	Cleaning in place
CMC	Critical micelle concentration
CMCL	Carboxymethyl cellulose
COD	Chemical oxygen demand
CTA	Cellulose triacetate
CTAB	Cetyltrimethylammonium bromide
CTAC	Cetyltrimethylammonium chloride
CTBD	Cooling tower blowdown
DAF	Dissolved air flotation
DBP	Disinfection byproduct
DCC	Dichloroisocyanurate
DCHP	Dicyclohexyl phthalate
DCP	2,4-Dichlorophenol
DEHP	Di-2-ethylhexyl phthalate
DEP	Diethyl phthalate
DMAc	N,N-Dimethylacetamide
DMF	N,N-Dimethylformamide
DMP	Dimethyl phthalate
DMSO	Dimethyl sulfoxide
DnBP	Dibutyl phthalate
DNP	2, 4-Dinitrophenol
DO	Dissolved oxygen
DOC	Dissolved organic carbon
DOM	Dissolved organic matter
DOP	Dioctyl phthalate
DP	Differential pressure
DTAB	Dodecyltrimethylammonium bromide
DVB	Divinylbenzene
E260	UV absorbance at 260 nm
ED	Electrodialysis
EDTA	Ethylenediaminetetraacetic acid
EEM	Excitation/emission matrix
EfOM	Effluent organic matter
EG	Ethylene glycol
EPS	Extracellular polymeric substances
ESCA	Electron spectroscopy for chemical analysis
FAC	Fibrous activated carbon
GFPD	Flat panel display
FRP	Fiber-reinforced plastic
FT-IR	Fourier transform infrared spectroscopy
GAC	Granular activated carbon
GC	Gas Chromatography
gfd	Gallons/ft^2^/day
GMA	Glycidyl methacrylate
GWRS	Groundwater Replenishment System
HA	Humic acid
HEM	n-Hexane Extractable Material
HLB	Hydrophilic–Lipophilic Balance
HPM	High-pressure membrane
HPO	Hydrophobic
HS-SPME	Headspace solid-phase microextraction
ICP	Inductively coupled plasma
ICR	Information Collection Rule
IEX	Ion exchange
IPA	Isopropanol
IR	Infrared
LAS	Linear alkyl benzene sulfonate
LbL	Layer-by-layer
LC-OCD	Liquid chromatography—organic carbon detection
LES	Lauryl ether sulfate
LMH	L/m^2^/h
LMW	Low molecular weight
LMWOCs	Low-molecular-weight organic compounds
log Dow	Logarithm of the pH-dependent n-octanol/water distribution coefficient
log *p*	Logarithm of the octanol-water partition coefficient
LOI	Loss on ignition
LP	Low pressure
LPM	Low-pressure membrane
MBR	Membrane bioreactor
MF	Microfiltration
MeOH	Methanol
MFI	Modified fouling index
MMF	Multi-media filter
MPD	m-phenylenediamine
MS	Membrane softening
MTCw	Water mass transfer coefficients
MW	Molecular weight
MWCO	Molecular weight cut-off
NBS	4-nitrobenzenesulfonyl chloride
NDMA	N-nitrosodimethylamine
NF	Nanofiltration
NMP	N-methyl-2-pyrrolidinone
NOM	Natural organic matter
NP	Nitrophenol
OCWD	Orange County Water District
ORP	Oxidation–reduction potential
O&G	Oil and grease
O&M	Operation and maintenance
PA	Polyamide
PAC	Powdered activated carbon
PALS	Positron annihilation lifetime spectroscopy
PAN	Polyacrylonitrile
PASP	Polyaspartic acid
PDMS	Polydimethylsiloxane
PEC	Polyelectrolyte complex
PEG	Polyethylene glycol
PEI	Polyethyleneimine
PEO	Polyethylene oxide
PES	Polyether sulfone
PFAS	Perfluoroalkyl substances
PFOS	Perfluorooctane sulfonate
PG	Propylene glycol
PHE	Phenol
pKa	Acid dissociation constant
PMSP	Poly(1-(trimethylsilyl)-1-propyne)
POE	Polyoxyethylene octyl phenyl ether
polyDADMAC	Polydiallyldimethylammonium chloride
PP	Polypropylene
PPA	Piperazine polyamide
PPCPs	Pharmaceuticals and personal care products
PPO	Polyphenylene oxide
PSF	Polysulfone
PSS-Na	Polystyrene sulfonate
PT-A	Polyvinyl methyl ether
PT-B	Tannic acid
PTFE	Polytetrafluoroethylene
PV	Pervaporation
PVA	Polyvinyl alcohol
PVAM	Polyvinylamidine
PVDF	Polyvinylidene fluoride
PVME	Polyvinyl methyl ether
PVP	Polyvinylpyrrolidone
RJA	Rejuvenation agent
RBSMT	Rapid bench-scale membrane test
RO	Reverse osmosis
SBS	Sodium bisulfite
SDBD	Sodium dodecyl benzenesulfonate
SDGs	Sustainable development goals
SDI	Silt density index
SDS	Sodium dodecyl sulfate
SEM	Scanning electron microscopy
SES	Swelling–embedding–shrinking
SGPW	Shale gas-produced water
SMPs	Soluble microbial products
SO	Sodium oleate
SOC	Synthetic organic compound
STPP	Sodium tripolyphosphate
SULP	Super ultra-low pressure
SUVA	Specific UV absorbance
SWRO	Seawater reverse osmosis
TBP	Tributyl phosphate
TCP	2,4,6-trichlorophenol
TDS	Total dissolved solids
TEP	Transparent exopolymer particles
TFC	Thin-film composite
TFN	Thin-film nanocomposite
TGA	Thermo gravimetric analysis
THM	Trihalomethane
TMC	Trimesoyl chloride
TMP	Transmembrane pressure
TOC	Total organic carbon
TPI	Transphilic
TSP	Trisodium phosphate
UF	Ultrafiltration
ULP	Ultra-low pressure
UPW	Ultrapure water
USEPA	US Environmental Protection Agency
XAD	Polymeric adsorbent resin
WBMWD	West Basin Municipal Water District
WF-21	Water Factory 21
WOCS	Weathered oil-contaminated seawater
